# Distribution and Differentiation of Wild, Feral, and Cultivated Populations of Perennial Upland Cotton (*Gossypium hirsutum* L.) in Mesoamerica and the Caribbean

**DOI:** 10.1371/journal.pone.0107458

**Published:** 2014-09-08

**Authors:** Geo Coppens d'Eeckenbrugge, Jean-Marc Lacape

**Affiliations:** 1 CIRAD, UMR 5175 CEFE, Campus du CNRS, Montpellier, France; 2 CIRAD, UMR AGAP, Montpellier, France; National Key Laboratory of Crop Genetic Improvement, China

## Abstract

Perennial forms of *Gossypium hirsutum* are classified under seven races. Five Mesoamerican races would have been derived from the wild race ‘yucatanense’ from northern Yucatán. ‘Marie-Galante’, the main race in the Caribbean, would have developed from introgression with *G. barbadense*. The racial status of coastal populations from the Caribbean has not been clearly defined. We combined Ecological Niche Modeling with an analysis of SSR marker diversity, to elucidate the relationships among cultivated, feral and wild populations of perennial cottons. Out of 954 records of occurrence in Mesoamerica and the Caribbean, 630 were classified into four categories cultivated, feral (disturbed and secondary habitats), wild/feral (protected habitats), and truly wild cotton (TWC) populations. The widely distributed three first categories cannot be differentiated on ecological grounds, indicating they mostly belong to the domesticated pool. In contrast, TWC are restricted to the driest and hottest littoral habitats, in northern Yucatán and in the Caribbean (from Venezuela to Florida), as confirmed by their climatic envelope in the factorial analysis. Extrapolating this TWC climatic model to South America and the Pacific Ocean points towards places where other wild representatives of tetraploid *Gossypium* species have been encountered. The genetic analysis sample comprised 42 TWC accessions from 12 sites and 68 feral accessions from 18 sites; at nine sites, wild and feral accessions were collected in close vicinity. Principal coordinate analysis, neighbor joining, and STRUCTURE consistently showed a primary divergence between TWC and feral cottons, and a secondary divergence separating ‘Marie-Galante’ from all other feral accessions. This strong genetic structure contrasts strikingly with the absence of geographic differentiation. Our results show that TWC populations of Mesoamerica and the Caribbean constitute a homogenous gene pool. Furthermore, the relatively low genetic divergence between the Mesoamerican and Caribbean domesticated pools supports the hypothesis of domestication of *G. hirsutum* in northern Yucatán.

## Introduction

Cotton (*Gossypium* spp.) is unique among crop plants in that four species have been independently domesticated in four different regions of the world: two tetraploids, *G. hirsutum* L. in Mesoamerica, *G. barbadense* L. in South America, and two diploids, *G. herbaceum* L. in Arabia and Syria and *G. arboreum* L. in the Indus Valley of India and Pakistan [1]. In the process, they were transformed from photoperiod-sensitive perennial sprawling or upright shrubs into short, compact, annualized day-length-neutral plants; and their small impermeable seeds sparsely covered by coarse, poorly differentiated hairs became larger and covered with abundant and long, white lint. Simultaneously, their seeds lost their impermeability and dormancy. The wide diversity of cotton results from the successive waves of agronomic improvement and human-mediated germplasm diffusion [1], [2].

Phylogenetic investigations in *Gossypium* distinguish 45 modern diploid species distributed among three major geographic lineages and eight genomes. The American tetraploid lineage originated within the last 1–2 million years from a single hybridization event between a maternal African A and an American D genome [1]. It diversified into five species, three wild endemic species, *G. darwinii* Watt native to the Galapagos, *G. tomentosum* Nutt. ex Seem. from the Hawaiian Islands, *G. mustelinum* Miers ex Watt restricted to Northeastern Brazil, and the two cultivated species *G. barbadense* and *G. hirsutum*. The latter provides over 90% of the world cotton, spreading North and South to subtropical and temperate latitudes well over 30° as an annual crop. Its indigenous (preindustrial) range encompasses most of Mesoamerica and the Caribbean, with two centers of morphological and genetic diversity, one in Southern Mexico-Guatemala, considered a primary center of diversity, and one in the Caribbean, where some introgression took place with *G. barbadense* [2]–[5].

In these two regions, *G. hirsutum* exhibits a diverse array of perennial forms, which Hutchinson [6] classified into seven geographical races. The primitive and highly variable race ‘punctatum’ is mostly found in Yucatán and round the coasts and islands of the Gulf of Mexico. Race ‘latifolium’ has a center of diversity in Guatemala and southern Mexico, but its range extends from most of Mexico to El Salvador and Nicaragua. Race ‘Marie-Galante’ is distinct both geographically and morphologically, with its pronounced apical dominance and tree-like habit. Its range includes the Antilles and Central America, South from El Salvador into northern to northeastern South America. Its origin and diffusion seems to be closely related to human migrations that would have resulted in the introduction of *G. barbadense* into Central America and the Antilles and its introgression with *G. hirsutum* in these areas [2], [3], [7], [8]. Together, these three most widespread races, ‘latifolium’, ‘punctatum’ and ‘Marie-Galante’, encompass most of the morphological variation in *G. hirsutum*. The remaining four races present a more restricted geographic distribution, with race ‘palmeri’ in the Mexican states of Oaxaca and Guerrero, race ‘morrilli’ in the central Mexican plateau, race ‘richmondi’ along the Pacific side of the Isthmus of Tehuantepec, and race ‘yucatanense’ limited to the northern coast of Yucatán. The latter is known only as a small, highly branched, sprawling shrub forming a dominant constituent of undisturbed beach strand vegetation. Hutchinson [6] considered race ‘yucatanense’ an extreme case of feral populations derived from primitive ‘punctatum’ landraces.

The persistence of wild populations of *G. hirsutum* has been the subject of considerable debate. On one hand, most germplasm collections came from man-made habitats, such as field plots and house yards, or highly disturbed habitats, such as roadsides and secondary vegetation, indicating that spontaneous cotton plants were escapes from cultivation. Furthermore, morphological differentiation appears similar and parallel for both landraces and feral plants [6], [9], [10]. Testing materials from Yucatán, Hutchinson [6] observed no differences between progenies of ‘punctatum’ from plants cultivated in dooryards and plants established in natural vegetation. On the other hand, Sauer [11] observed that the northern Yucatán wild cottons are negatively associated with human settlements and form a dominant constituent of “a complex vegetation type occupying a coherent and extensive area with natural and edaphic and climatic boundaries.” He maintained this interpretation in his study of the Cayman Islands shoreline vegetation [12].

In a study of the effects of domestication in *G. hirsutum*, Stephens [9] extended the question to the seemingly wild populations of race ‘punctatum’ observed on the dry leeward sides of some of the Greater Antilles, on Florida Cays [6], [13], along the coasts of the Gulf of Mexico as well as in Venezuela. For a long time, he could not rule out the possibility that these forms are feral relics of pre-Columbian or early post-Columbian cultivation [9], [14], even though they have retained their small impermeable seeds with an impressive capacity for long distance dispersal [14], [15]. Only from 1967 did he abandon the views of Hutchinson et al. [16] and refer without restriction to coastal populations in the Caribbean and the Gulf of Mexico as wild [3], [7]. In their extensive collecting travels, Ano et al. [17], Ano and Schwendiman [18], and Schwendiman et al. [19] went even further in underlining the similarity of these cotton populations with those of northern Yucatán shores, relating their distribution to sea currents, and classifying them in the same race ‘yucatanense’.

Long-range seed dispersal also explains the presence of *G. hirsutum* in the Pacific Ocean. Fryxell and Moran [20] described a truly wild small ‘punctatum’ population in Socorro, an island of the Revillagigedo archipelago, some 600 km West of Mexico. Similar wild forms have diffused to even more distant Pacific islands (Tahiti, Marquesas, Samoa, Fiji, and Wake islands) [20]–[22]. Indeed, Fryxell [23] suggested a close relationship between the evolutionary history of the tetraploid cotton species and their particular adaptation to strand habitats along marine beaches, underlining the importance of oceanic seed diffusion and citing a dozen cases of such populations, eight of which concerned *G. hirsutum*. He presented a hypothesis relating this coastal adaptation and capacity for diffusion via ocean currents to the significant mobility of shorelines during the Pleistocene. In 1979, Fryxell further developed his views in his monograph on the Malvaceae [24], adding to his arguments those of Sauer [11]. Since then, the question of the natural dispersion of *G. hirsutum* has been further complicated by the recent description of wild populations of *G. hirsutum* in Paraguay [25], confirming an intuition of Stephens [7].

Despite its importance for cotton genetics and breeding, the question of truly wild cottons has spawned relatively few genetic studies. In their RFLP study, Brubaker and Wendel [8] observed three groups: (1) races ‘yucatanense’ and ‘punctatum’, (2) races ‘latifolium’ and ‘palmeri’, and (3) race ‘Marie-Galante’. They refuted Hutchinson's views on the regressive status of race ‘yucatanense’, and proposed a model where “the morphological intergradation, geographical proximity, and genetic similarity of race ‘yucatanense’ to inland ‘punctatum’ populations – of Yucatán – reflects a relationship between the first domesticated form of *G. hirsutum* and its wild progenitor.” Thus, the initial stages of cotton (*G. hirsutum*) domestication would have taken place in northern Yucatán and the human-mediated transfer of the first ‘punctatum’ cottons out of the species' natural range would have triggered the process of concomitant differentiation into new and improved races, agronomic developments, and long range germplasm diffusion. This process would explain the current distribution of *G. hirsutum* diversity. The SSR study of Lacape et al. [26] supported the racial classification [6], and the interracial relations appeared consistent with the model of progressive domestication, diffusion and differentiation proposed by Brubaker and Wendel [8], except for the geographically more distant ‘Marie-Galante’, which appeared closely related to ‘punctatum’. Their three ‘yucatanense’ accessions from Guadeloupe (as classified by Ano et al. [27]) exhibited a high number of unique alleles. Similarly, in the study of Liu et al. [28], the unique representative of race ‘yucatanense’, from Yucatán, appeared highly divergent from the other accessions.

The views of Brubaker and Wendel [8], which explain the pre-Columbian *G. hirsutum* diversity by successive waves of diffusion of genetic and agronomic developments, from northern Yucatán to inland Yucatán (race ‘punctatum’), then to southern Mexico and Guatemala (race ‘latifolium’), and finally to all Mesoamerica and the Caribbean, imply an early cotton domestication. This is consistent with the contributions of historical linguistics and archaeology. Thus, words for cotton can be reconstructed in Proto-Otomangue, a language that was spoken in Central Mexico at least 6500 BP [29], [30]. According to Smith and Stephens [31], the oldest remains of Mesoamerican cotton, found in the Tehuacán Valley and dated 5500 to 4300 BP, represent fully domesticated introductions, being comparable in form and size to the landraces currently existing in the same area.

The domestication and diffusion scenario proposed by Brubaker and Wendel [8] for *G. hirsutum* has been generally accepted and it is found practically unchanged in the most recent syntheses [1], [32]. The fact that it is based on only one wild population (from northern Yucatán) has not been challenged, and alternative scenarios have been overlooked. Nevertheless, as stated by Sauer [11], “if lint bearing cottons were naturally present in the New World as sea dispersed pioneers, they were not likely to be confined to Yucatán… The lint may have been widely gathered and perhaps traded long before regular cultivation began; the process of domestication may have been diffuse in space and time, involving wild cottons from Caribbean and Pacific coasts, as well as Yucatán.” Indeed, if *G. hirsutum* is a perennial whose regressive forms thrive in disturbed human habitats and xerophytic secondary vegetation, domestication was not necessarily a linear process, moving from littoral strands to the agricultural field through the dooryard. Wendel et al. [4] questioned “whether *G. hirsutum* achieved widespread distribution and regional differentiation as a wild plant prior to domestication, or if it was widely distributed as a perennial semi-domestic by the pre-Columbian people from a much smaller native range”. Casas et al. [33] have described how Mesoamerican societies have improved more than 200 plant species, through management practices that integrate cultivated areas, agroforestry systems and gathering from the wild, with or without conscious selection. As documented from many studies of cactus fruit species [34]–[36], the result of this *in situ* domestication process is a mosaic of habitats and useful plant populations with particular morphological, genetic, and even reproductive characteristics, according to management intensity. Similar management practices may have been used for cotton. Stephens [9] cites several accounts, from the first voyage of Columbus to much later periods in colonial times, mentioning the simultaneous exploitation of cultivated, feral and wild cottons, according to the quality objective.

If we recognize that Caribbean wild cotton populations may have been involved in the domestication process, we must also question the distinction between a primary centre of diversity in Mesoamerica and a secondary centre in the Caribbean. The strong dominance of race ‘Marie-Galante’ in the latter region, as well as in southern Central America and northern South America, poses the question of its origin and even of its possible separate domestication [7], [32].

The question of the natural distribution of *G. hirsutum* is not only crucial for understanding the biogeography of tetraploid cottons, and their evolution and diffusion under domestication, but also for the continuation of the domestication process. Further improvement of the crop requires both a better exploitation of the available germplasm and better genetic tools to manipulate important economic traits [32]. For example, studies on the effects of domestication on such essential traits as fiber development [37] and the corresponding genetic transformations, with an altered expression of about 25% of the genes at transcriptome level [38], depend on the comparison of well-defined and representative samples of wild and domesticated germplasm.

We present here a double approach to investigate this question, combining Ecoclimatic Niche Modeling (ENM) and neutral genetic markers to assess whether coastal cotton populations are “truly wild,” and investigate their relationship with inland perennial cottons. ENM methods derive an envelope for the environmental requirements of a taxon from a set of its occurrence localities. They have provided a powerful tool for investigating the ecology and distribution of both plant and animal species. An ENM study on *G. hirsutum* was recently published by Wegier et al. [39] aiming to understand not only the distribution of “wild” cotton populations from Mexico, but also the spatial organization of genetic diversity and potential gene flow from genetically modified cultivars using molecular markers. However, as compared to the wild cotton studies cited above, Wegier et al. [39] used much more permissive criteria to distinguish feral and wild cottons. In our approach, we have used ENM to document the relationship between perennial cotton domestication and distribution in Mesoamerica, the Caribbean and the Gulf of Mexico, by mapping and comparing the potential tropical/subtropical distributions of domesticated *G. hirsutum* populations, feral cotton (escaped from cultivation), and presumably or truly wild populations of races ‘yucatanense’ and ‘punctatum’. Potential distribution of *G. hirsutum* was predicted for modern climatic conditions as well as for climatic parameters modeled for the Last Glacial Maximum (LGM). The underlying idea is that the original distribution of wild cotton in the early Holocene was necessarily related to its distribution during the Pleistocene, following an approach validated by several studies [40], [41]. The identification of potential climatic refuges for the species should help in distinguishing natural and human factors in its dispersal.

As the ENM study confirmed the particular ecology and “truly wild” status of a number of coastal cotton populations, SSR neutral genetic markers were then used to characterize them and investigate their relationship with neighboring feral cottons.

## Materials and Methods

### Climatic modeling and analysis

Our ENM study focused on the centers of diversity of *G. hirsutum*, i.e., Mexico, Central America, and the Eastern Caribbean (from the coasts of Venezuela to Florida through the Antilles). From now on, we will collectively refer to this region as Mesoamerica and the Caribbean. Geographical and ecological information was extracted from the CIRAD cotton germplasm database and records [17]–[19], [27], [42], [43] and related collecting reports by French and US scientists (collections in the 80s under the aegis of the former IBPGR), the scientific literature on wild cotton, regional floras, herbarium-label and germplasm-passport data obtained from the Global Biodiversity Information Facility (GBIF) portal, the Mexican Red Mundial de Información sobre Biodiversidad (REMIB), and relevant Mexican administrative documents. All geographic coordinates have been assigned or verified against associated geographic information with gazetteers (mostly Google Earth and Geonames). Incomplete or imprecise records were discarded, as were redundant data (dataset available upon request).

The information associated with the collections/observations was also used to classify cotton occurrences according to their status on a wild to cultivated scale, using four categories: ‘cultivated’ (fields and dooryards), ‘feral’ (plants found in disturbed habitats, such as roadsides and secondary vegetation), ‘wild/feral’ (plants found in preserved habitats and/or forming persistent populations), and ‘truly wild’ (populations described as such by experts, based on ecological and morphological grounds). This categorization is partially analogous to that used by Stephens [9], whose “wild forms” would include both our ‘truly wild’ and ‘wild/feral’ categories, whereas Stephens’ “semiferal” and “commensal/cultivated” forms correspond to our ‘feral’ category and cultivated categories, respectively. The objective was also analogous: Stephens tested his categories on domestication traits (fiber and seeds) while we aimed at testing them on eco-climatic grounds.

For each occurrence record, 19 bioclimatic variables were extracted from WorldClim, a package consisting of global surfaces of climate, with a 2′30″ grid resolution (corresponding roughly to 4.4×4.6 km) [44]. These variables are: 1) annual mean temperature; 2) mean diurnal range (mean of monthly (max temp - min temp); 3) isothermality (Bio2/Bio7); 4) temperature seasonality; 5) maximal temperature of warmest month; 6) minimal temperature of coldest month; 7) temperature annual range; 8) mean temperature of wettest quarter; 9) mean temperature of driest quarter; 10) mean temperature of warmest quarter; 11) mean temperature of coldest quarter; 12) annual precipitation; 13) precipitation of wettest month; 14) precipitation of driest month; 15) precipitation seasonality; 16) precipitation of wettest quarter; 17) precipitation of driest quarter; 18) precipitation of warmest quarter; and 19) precipitation of coldest quarter.

For ENM, we chose the widely used Maxent machine learning method. It estimates the probability distribution of maximum entropy (i.e. closest to uniform) subject to the constraint that the expected value of each environmental variable (or its transform and/or interactions) under this estimated distribution matches its empirical average [45]. Maxent was run twice, firstly on the whole dataset, and secondly only on points in the ‘truly wild’ category. A logistic threshold value equivalent to the 10 percentile training presence was retained to separate climatically favorable areas from marginally fit areas. Maxent output provides measures of the contribution of each bioclimatic variable (percent contribution and permutation importance) and proposes a jackknife test to quantify the contribution of each variable from the gain when it is used in isolation and the gain loss when it is omitted from the model. However, the strong correlations among bioclimatic variables do not allow an easy interpretation of their relative importance. Therefore, we performed a principal component analysis (PCA) to characterize and compare the climatic envelopes of our categories of *G. hirsutum* observations, discarding those variables whose contribution appeared marginal. The factors with an eigenvalue above 1 were retained and a normalized varimax rotation was applied to maximize the sum of the variances of the squared loadings, simplifying the interpretation of the results. The different categories of populations were then plotted on the principal components plane to visualize and compare their ecoclimatic range.

To predict the potential distribution of *G. hirsutum* at LGM, the MIROC climatic model [46] derived from the PMIP2 database Paleoclimate Modelling Intercomparison Project Phase II for 21,000 BP was downloaded from the Worldclim website (http://www.worldclim.org/) and used on a dataset restricted to the ‘truly wild’ category.

### Genetic analyses

**Figure 1:**
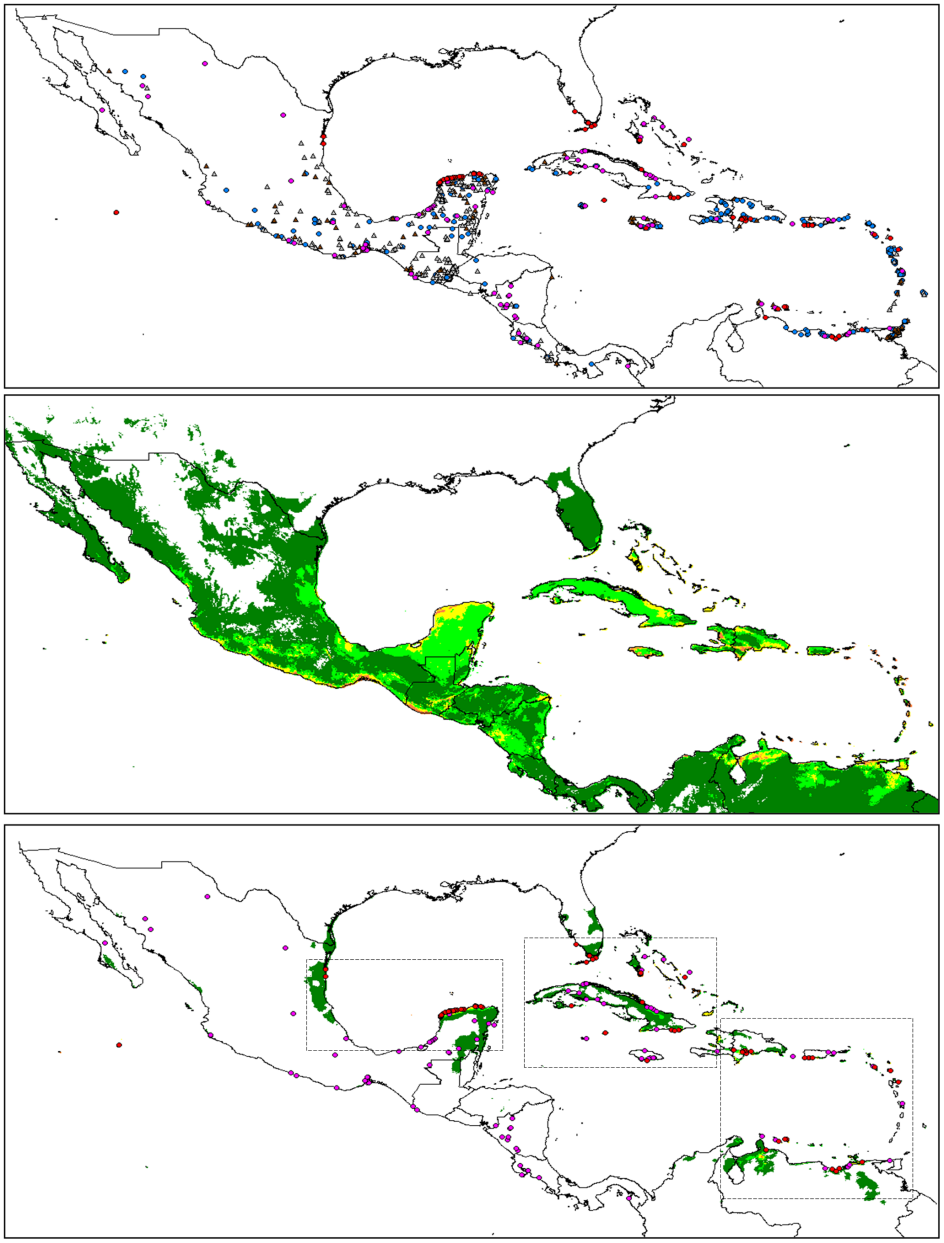
Distribution and climate model of perennial forms of *G. hirsutum* in Mesoamerica and the Caribbean. **A.** Distribution of 954 categorized datapoints for perennial forms of *G. hirsutum* in Mesoamerica and the Caribbean ‘truly wild’ (TWC) specimens/populations are represented by red dots, ‘wild/feral’ by purple dots, ‘feral’ (disturbed habitats) by blue dots, ‘cultivated’ by brown triangles, and unclassified plants by grey triangles. **B.** Climate model for distribution of both cultivated and spontaneous *G. hirsutum* in Mesoamerica and the Caribbean (complete set as presented in Figure 1A). Climate suitability is indicated by background color from unfavorable (no color) to marginal (dark green) or increasingly favorable (light green and warmer colors). **C.** Localization of the populations from categories TWC and ‘wild/feral’ and climate model for TWC populations. Red dots represent the datapoints used for the distribution model (TWC populations), whereas purple dots represent ‘wild/feral’ populations of uncertain status (truly or secondarily wild). Climate suitability is indicated as in Fig. 1B. Three dotted frames refer to map limits as magnified in Figure 2.

The panel of accessions of perennial *G. hirsutum* cotton populations used for SSR genotyping comprised 110 feral and wild accessions supplemented by a modern cultivar, ‘FM966’ (Table 1). One hundred and eight accessions originated from the CIRAD seed bank, and three from USDA. Twenty-nine countries/provinces of Mesoamerica and the Caribbean were represented (Table 1). Particular attention was paid to geographic locations where both truly wild and feral populations could be identified in close proximity (such as for the populations of Pointe des Châteaux in Guadeloupe), or slightly more distant (such as for the populations from Yucatán sea-shores versus inland). Such sites with both truly wild and nearby feral specimens were identified in nine cases (Mexico/Yucatán, Jamaica, Dominican Republic, Puerto Rico, St Kitts & Nevis, Guadeloupe, Venezuela, Bonaire, and Curaçao). Three localities were represented only by wild specimens, Florida (one feral specimen discarded due to missing data), Antigua, and Socorro Islands of Mexico; and 18 additional localities were only represented by feral populations. A few additional locations where truly wild cotton (further abbreviated as TWC) populations had been reported (visible as red dots in Figure 1) could not be included in the genetic study due to lack of plant material, such as in Cuba, the western coast of the Gulf of Mexico (Tamaulipas), Bahamas and Grand Cayman. Detailed geographic information of the 110 accessions is available in Table S1; Figure S1 presents their localizations on the sites with TWC populations.

**Table 1:** Repartition among races of *G. hirsutum* of the 110 selected accessions per country of origin and type/race (Nota: 9 locations had both ‘truly wild’ (TWC) and feral specimens).

		Feral					Total feral	Total wild	Total
Country race:	MG	MO	PA	RI	PU	unk		TWC	
Antigua & Barbuda								1	1
Aruba	1						1		1
Bahamas	1						1		1
Barbados	1						1		1
Bonaire	2						2	2	4
BWI_Grand Cayman	1						1		1
Colombia	2						2		2
Costa Rica	1						1		1
Cuba	1						1		1
Curaçao	4						4	4	8
Dominican Rep	4						4	4*	8
Dominica	1						1		1
French Guiana	1						1		1
Guadeloupe	6						6	4	10
Guam Pacific					1		1		1
Guatemala	1						1		1
Haïti	1						1		1
Jamaica	3						3	1	4
Maldives					1		1		1
Martinique	1						1		1
Mexico		1	1	1	9		12	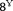	20
Nicaragua	1						1		1
Puerto Rico	8						8	7†	15
Saint-Kitts & Nevis	2						2	3	5
Saint-Vincent & Grenadines	1						1		1
Samoa						1¶	1		1
Trinidad & Tobago	2						2		2
USA-Florida								3	3
Venezuela	7						7	5	12
**Total** ‡	**53**	**1**	**1**	**1**	**11**	**1**	**68**	**42**	**110**

Races are referred as MG for ‘Marie-Galante’, MO for ‘morrilli’, PA for ‘palmeri’, PU for ‘punctatum’, RI for ‘richmondi’, unk for ‘unknown’. TWC collectively refers to truly wild cotton populations including those of race ‘yucatanense’ from Mexico (see text).

* = also referred-to as *G. ekmanianum*,

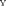
 = 8 accessions from Mexico include 7 accessions from Yucatán and one from Socorro Islands,

† = local name “algodon brujo”,

‡ = one additional accession from Australia (FM966) as modern cultivated (total = 111),

¶ = accession initially of «unknown» race (further assigned as race ‘punctatum’).

Five seeds per accession were sown in small pots in the greenhouse in Montpellier and DNA was extracted from pooled samples (1–3 different plants) of young leaves using the MATAB protocol [47]. Thirty-seven SSR markers were selected for genotyping based on previous experience [26], in order to optimize information and quality. They were mostly derived from non-coding genomic DNA sequences (majority from series ‘BNL’ and ‘CIR’), preferably to the more frequent EST-derived SSRs, with presumptive neutrality (no evidence of having been targeted during domestication). They had shown in previous experiments the amplification of a single PCR product in tetraploid cotton, thus avoiding the ambiguity generated by homoeolog loci. SSRs were genotyped in multiplex panels of 8 SSRs (four dyes and two SSRs per dye). Simultaneous PCR amplifications in a final volume of 10 µl contained 5 ng of genomic DNA, 200 µM of each dNTP, 0.5 mM MgCl2, 1 U Taq polymerase, 0.08 µM of M13-tailed ‘F’ primer, 0.1 µM of both the ‘R’ primer and of an M13 oligonucleotide tailed with the ad hoc fluorochrome. PCR reactions were performed on an Eppendorf microcycler (Eppendorf, Madison, WI)) using the following profile, a hot start of 94°C for 5 min, 35 cycles of 30 sec at 94°C, 1 min at 55°C and 1 min at 72°C, and a final extension step of 30 min at 72°C. PCR products were pooled with 10 µl of GeneScan 600-LIZ size standard. PCR products were denaturated and size fractionated using capillary electrophoresis on an ABI 3500 Genetic Analyzer (Applied Biosystems). Subsequently, GeneMapper 4.1 (Applied Biosystems) software was used for allele size estimation.

Twenty-six SSRs showing strict and unambiguous bi-allelic patterns (coded as homozygote when a single peak/allele and heterozygote with 2 peaks/alleles) were selected. The 26 SSRs were mapped on 18 of the 26 chromosomes (Table S2). Expected heterozygosity at each locus was calculated as *He* = 1−Σpi^2^ where pi is the frequency of the ith allele.

The data matrix of bi-allelic codings for the 26 SSRs and 111 genotypes was imported into the DARWin5 software [48] to calculate genetic dissimilarities. Bootstrap dissimilarity matrices were calculated by drawing 10 000 entries. A Principal Coordinate Analysis based on the similarity matrix was conducted also with DARWin package. In complement to this factorial analysis, unweighted trees without topological constraints were constructed using a neighbor joining (NJ) approach [49] to represent individual relations. Lastly, the methods implemented in the STRUCTURE software [50] were used to infer population clusters and estimate admixture (quantitative clustering). The number of clusters, K, was chosen based on 20 independent runs for K values ranging between 1 and 5 with a burn-in length of 500,000 followed by 750,000 MCMC iterations. The ΔK method [51] was then applied using Structure Harvester [52], and estimated membership for each genotype, in each cluster, was read from the STRUCTURE output.

## Results and Discussion

### Dataset composition and distribution for climatic modeling

A total of 954 datapoints were gathered, of which 630 could be ascribed to our four categories (Table 2). Figure 1A shows no clear differences in the distributions of the different categories, except for ‘truly wild’ cotton (TWC) populations, which only occur along the coasts of the Eastern Caribbean and the Gulf of Mexico. The sample is well balanced between Central America and Mesoamerica, on one hand, and the islands and shores from the Eastern Caribbean to Florida on the other hand. Feral and wild specimens are better represented than cultivated germplasm, which can be explained by a collecting bias of botanists, most often interested by spontaneous plants, and germplasm collectors, motivated by the rusticity expected from primitive and spontaneous materials. The poor representation of ‘cultivated’ cotton also reflects the decline of its cultivation in Mexico [10] and in the Caribbean [18].

**Table 2:** Dataset composition and distribution among domestication status categories of perennial *G. hirsutum* as defined for the present study.

	Total	Uncategorized	cultivated	feral	wild/feral	truly wild
Meso- & Central America	544	308	61	80	59	36
Eastern Caribbean to Florida	410	16	96	188	46	64
Total	954	324	157	268	105	100

Among the 544 datapoints from Central and Mesoamerica, few have been assigned to a geographical race: 2 for race ‘morrilli’ (state of Guerrero), 8 for ‘palmeri’ (Guerrero), 5 for ‘richmondi’ (Oaxaca), 41 for ‘punctatum’ (Yucatán peninsula and Socorro Island), and 32 for ‘yucatanense’ (state of Yucatán). Albeit poor, this information is consistent with their original description by Hutchinson [6] and, with the exception of ‘yucatanense’, all races are found in both ‘cultivated’ and ‘feral’ categories, illustrating the absence of morphological differentiation between cotton landraces and feral cottons within a same region, as reported by several collectors [9], [10], [24]. ‘Punctatum’ is the only race with important spontaneous populations classified as ‘wild/feral’, one in the state of Yucatán, around Celestún, and several ones on the southern coast of Campeche state, between Champotón and Isla del Carmen. The only ‘truly wild’ Mexican population of race ‘punctatum’ is the one described by Fryxell and Moran [20] in the Socorro Island (Revillagigedo archipelago).

For the Eastern Caribbean (410 accessions from Venezuela to Florida), most observations were from breeders, so the racial composition is much better documented. It shows a strong dominance of race ‘Marie-Galante’ (278 acc.). The only other identified race is ‘punctatum’, ascribed to the TWC category (64 datapoints) or, exceptionally, to the ‘wild/feral’ category (one datapoint). In our dataset, these TWC are classified as ‘punctatum’, following the early views of Hutchinson [13], author of the original classification, although the same materials collected by Ano et al. [27] and Schwendiman et al. [19] were later reclassified under race ‘yucatanense’.

### Ecoclimatic niche models for cultivated, feral, and wild *G. hirsutum*

Figure 1B presents the potential distribution extrapolated by the Maxent software for the whole dataset. Along the coasts of Mexico, climatically favorable lowland areas correspond to those identified by Wegier et al. [39], i.e., the Yucatán peninsula, the regions of Veracruz and Tamaulipas along the western shores of the Gulf of Mexico, and the tropical Pacific coast. The latter area appears particularly favorable. The state of Tabasco (southern shores of the Gulf of Mexico) is better represented than in the study of Wegier et al. [39]. Other favorable areas are found much further inland.

Given the relative over-representation of wild and feral materials in our sample, Figure 1B gives a likely picture of the Mesoamerican distribution of perennial *G. hirsutum* for the last three millennia at least, i.e. a period of very active agricultural development, during which modern climatic conditions were already established [53]. The distribution of favorable areas corresponds quite well with those areas where several of Hutchinson's geographic races were developed: Yucatán to Mexican shores of the Gulf of Mexico for race ‘punctatum’, Yucatán to Guatemala for race ‘latifolium’, Pacific regions and the southern side of the isthmus of Tehuantepec for races ‘palmeri’ and ‘morrilli’, and even regions of the central Mexican plateau for race ‘richmondi’. In Central America, the pre-Columbian distribution of *G. hirsutum* appears related to the diffusion of race ‘Marie-Galante’, as the favorable areas close to the Guatemalan-Salvadoran border and in western Nicaragua show good correspondence with the distribution of this race, presented by Stephens [7]. As suggested by this author, these races probably differentiated under relative geographical, ecological and cultural isolation, the latter term covering “the combined effects of human selection, migration and diffusion.”

**Figure 2:**
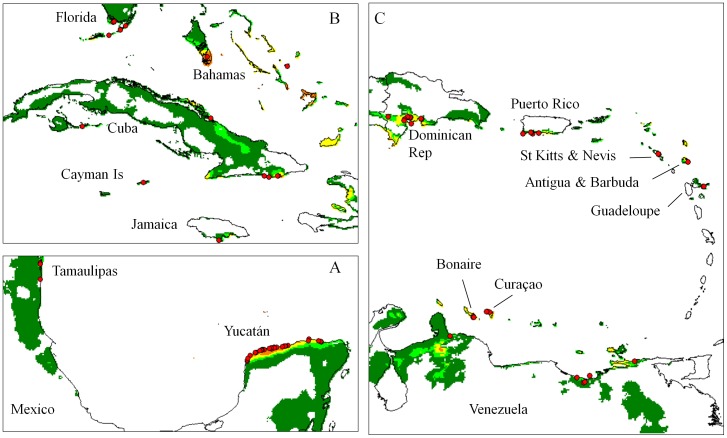
Localization of the truly wild cotton (TWC) populations and corresponding climate model. Map frames indicated as rectangles in Figure 1C. Climate suitability as indicated in Fig. 1. **A.** Gulf of Mexico. **B.** Florida and western Greater Antilles. **C.** Venezuela and eastern Caribbean.

Figure 1C presents the geographical distribution of ‘wild/feral’ and TWC populations, together with a distribution model based only on ‘truly wild’ populations (100 datapoints). The areas suitable for TWC populations (Figure 1C) cover a very small part of the favorable areas for the whole sample (Figure 1B). They are mostly found in three sub-regions: (i) Gulf of Mexico and northern Yucatán, (ii) Florida and western Greater Antilles and (iii) Venezuela and eastern Caribbean, as detailed in Figures 2A, B and C, respectively.

The great majority of ‘wild/feral’ populations (purple dots on Figure 1C) fall in areas that are marginal (dark green areas) or unsuitable for TWC populations, validating our a priori categorization.

The ‘yucatanense’ population along the northern coast of Yucatán (Figure 2A), certainly constitutes the most extensive TWC population [43], [54]. Our model confirms that its distribution is clearly limited by ecological parameters, as stated by Sauer [11]. Within this well-delimited area, a few specimens classified as ‘wild/feral’ are very probably incompletely documented representatives of race ‘yucatanense’. Extensive spontaneous populations also exist on the western coast of the Yucatán peninsula, but we have found no indications that these are ‘truly wild’. On the contrary, the model indicates that they have developed under climatic conditions that are not even marginally fit for TWC populations. West of the Gulf of Mexico, along the coast of Tamaulipas, ‘truly wild’ *G. hirsutum* was observed by Lukefahr cited in Stephens [7]. However, favorable areas are small and sparse in this region, and we could trace only three specimens whose labels mention that they were parts of natural coastal vegetation. Confirming the statement of Stephens [7], no population that could be classified as TWC has been documented for the Pacific coast of Mexico, where a very few small coastal areas appear climatically marginal for sustaining such populations. Thus, while the model confirms highly favorable climatic conditions in the Revillagigedo Islands, it gives no clear indication about areas where wild *G. hirsutum* could have developed on the western coast of Mexico before diffusing to islands in the Pacific Ocean.

In northern South-America and the southern Caribbean (Figure 2C), TWC populations are scattered along the coasts of Venezuela, between the Gulf of Venezuela (Saco de Maracaibo; state of Falcón) and the North of the state of Sucre, and on the shores of many islands along these coasts: Curaçao, Bonaire, Isla de Piritú. We have found only ambiguous information for the Chacachacaré Island. Mentions of colonial cotton plantation cast doubt on the only report of wild cotton populations in this area by Stephens [9]. On the other hand, the surroundings of Chacachacaré village in the Island of Margarita offer excellent conditions for TWC populations, suggesting that the homonymy of these neighbor sites may have created confusion. To the West, the shores of Colombia only offer marginal conditions for TWC (Figure 1C), which explains why Stephens [9] was not successful in his search for wild cotton in this area. To the Northeast of Venezuela, there seems to be another gap in the natural distribution of *G. hirsutum*, as no TWC populations have been identified in Trinidad and Tobago or in the southern half of the Lesser Antilles (Figure 2C), which is consistent with the descriptions of Hutchinson [13], [55]. In the northern Lesser Antilles (Figure 2C), only three TWC populations have been described, in Guadeloupe [27], in Antigua and in Saint Kitts [9], [18], [19], and the model confirms favorable climatic conditions at these sites.

In the Greater Antilles (Figure 2B and 2C), the modeled distribution also agrees well with the wealth of previous reports of TWC populations of race ‘punctatum’, indicating favorable climatic conditions for the “algodón brujo” of southern Puerto Rico [9], [13], [19], for the populations around the Yaquí Valley of the Dominican Republic [9], [19], [56], in Haiti [13], Jamaica [19], [57], [58] and in the Cayman Islands [12], [59], [60]. In southern Cuba, similar populations exist around Guantánamo (specimen labels refer to the morphological type described by Britton in 1908 [57]). Further North, the modeled distribution is consistent with the observations of TWC in Florida [19], [23] and in the Bahamas [61], [62]. For Bermudas, much further North, the model indicates unfavorable conditions for TWC, which is consistent with the statement by Britton [63] about the absence of native cotton in these islands.

### Climatic requirements of cultivated, feral and wild populations of perennial cotton

Seven variables were discarded for PCA on climatic variables, because of their poor specific contribution to the Maxent model obtained from the whole sample: isothermality (Bio3), maximal temperature of the warmest period (Bio5), precipitation of the wettest and driest periods (Bio13 and Bio14), precipitation seasonality (Bio15), and precipitation of the driest and warmest quarters (Bio17 and Bio 18).

**Figure 3:**
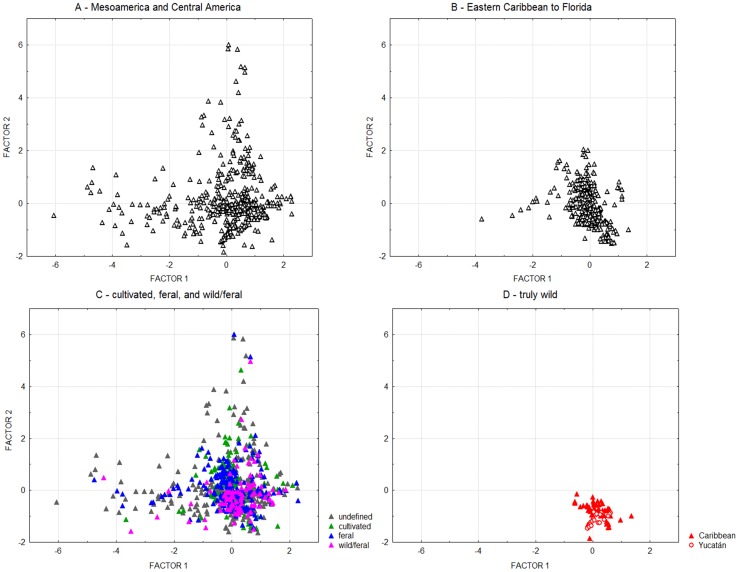
Principal component analysis of *G. hirsutum* climatic envelope. Climate variables are listed in Table 3. Comparison of different subsamples in Mesoamerica, the Eastern Caribbean and Florida, according to origin (A, B) and domestication status (C, D).

The analysis on the remaining twelve variables produced three factors with an eigenvalue superior to 1 (Table 3). The first one is strongly associated with mean temperatures at all periods of the year (Bio1, and Bio8-11), with correlations between 0.82 and 0.95; the second one is associated with precipitation (Bio12, 16 and 19), with correlations between 0.80 and 0.95; and the third one is associated with variables related to latitude (Bio2-7: diurnal temperature range, temperature seasonality, minimal temperature of coldest period and temperature annual range). The third factor shows no clear differences among our categories, which is consistent with their similar latitudinal dispersion, from tropical Venezuela to subtropical northern Mexico and Florida. In contrast, the categories and origins present different patterns of dispersion in the plane formed by the two first principal component factors (Figure 3). On the continent (Central and Mesoamerica, Figure 3A), part of the observations come from cooler regions (along the x-axis of factor 1, to the left) or from wetter regions (along the y-axis of factor 2, upwards), while cotton-associated climates appear more uniform in the eastern Caribbean (Figure 3B). *G. hirsutum* was not observed in regions that are both cooler and wetter (upper left area in Figure 3), which gives the general shape of an inverted ‘L’ to the Mesoamerican dot cloud.

**Table 3:** Principal component analysis (Varimax normalized rotation) on a set of bioclimatic variables retained for their contribution to the Maxent ecoclimatic model of distribution: factor loadings (values higher than 0.70 in bold characters).

Variable	Factor 1	Factor 2	Factor 3
1-Annual mean temperature	**0.93**	0.04	0.33
2- mean diurnal range	−0.06	0.04	**−0.82**
4- temperature seasonality	−0.01	−0.29	**−0.80**
6- minimal temperature of coldest month	0.54	0.12	**0.83**
7- temperature annual range	−0.07	−0.09	**−0.97**
8- mean temperature of wettest quarter	**0.90**	−0.18	−0.03
9- mean temperature of driest quarter	**0.82**	0.17	0.37
10- mean temperature of warmest quarter	**0.95**	−0.11	−0.15
11- mean temperature of coldest quarter	0.68	0.18	0.63
12- annual precipitation	−0.07	**0.95**	0.14
16- precipitation of wettest quarter	−0.00	**0.95**	−0.03
19- precipitation of coldest quarter	0.02	**0.80**	0.21
Proportion of total variance	0.34	0.22	0.31

When considering domestication status, no clear distinction can be made between ‘cultivated’, ‘feral’, and ‘wild/feral’ materials (Figure 3C), as these categories share the same general inverted ‘L’ pattern of dispersion in the principal components plane. In contrast, TWC populations are clearly characterized by very uniform climatic conditions; thus the environment of both ‘yucatanense’ and truly wild ‘punctatum’ (Figure 3D) is clearly among the hottest and driest in our sample. The best represented geographical race, ‘Marie-Galante’, which is highly dominant throughout the Antilles, logically presents the same climatic dispersion as the general Caribbean sample, with occurrences under extremely arid conditions too (not shown). Indeed, several reports mention spontaneous ‘Marie-Galante’ populations in the vicinity of TWC populations, as in Puerto Rico [13], Saint Kitts [18], and Guadeloupe [27].

### Potential distribution of native *G. hirsutum* in America and the Pacific

**Figure 4:**
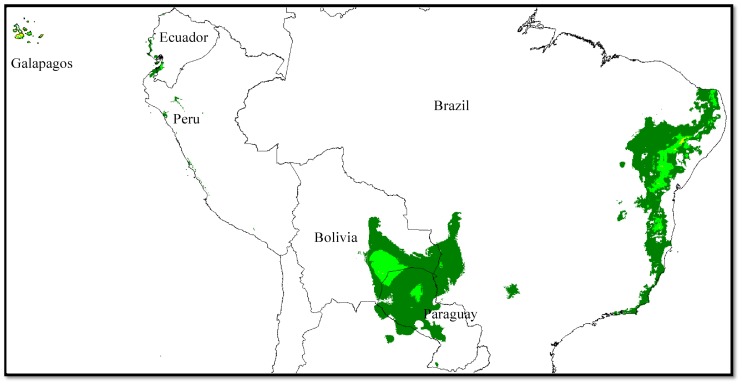
Potential distribution of truly wild *G. hirsutum* in South America. Distribution as extrapolated from the climate model presented in Figures 1C and 2.

Both the ENM and factorial analyses clearly show that TWC populations of *G. hirsutum* present an exceptional combination of a narrow environmental niche and a highly geographically scattered distribution. Stephens [64] has related the capability for long distance dispersal of tetraploid cotton seeds to their buoyancy and tolerance to prolonged immersion in salt water. It is therefore interesting to extend the TWC climatic model derived from occurrences in Mesoamerica and the Caribbean to a larger area in South America and the Pacific. Figure 4 presents the results of this extrapolation in South America. Four areas offer favorable climatic conditions, two inland areas, Bolivia/Paraguay and Northeastern Brazil, and two coastal areas, Ecuador/Peru and Pacific islands. Strikingly, all of them are validated by the existence of wild populations of tetraploid cottons. The favorable area in Bolivia and Paraguay was suggested long ago by Stephens [7] and, indeed, a wild form of *G. hirsutum* has been reported there recently [25]. Its inland situation renews the question of tetraploid cotton dispersal, as it implies non-oceanic diffusion. A bird-related mechanism is the likely explanation [15]. The other potential inland area, in Northeastern Brazil, corresponds well to the distribution of *G. mustelinum*, a wild tetraploid endemic to the region [65]–[67]. The third area, in the arid coastal regions of southern Ecuador and northern Peru and in the Galapagos Islands, corresponds with the distribution of 2 other wild tetraploid *Gossypium species*: (i) the wild populations of *G. barbadense* (North and South of the Guayas estuary) and (ii) the wild tetraploid species *G. darwinii*, a close relative of *G. barbadense*, endemic to the Galapagos islands [68].

In the fourth favorable area (not shown), further west into the Pacific, Worldclim coverage is incomplete, particularly for small atolls, so all climatically suitable sites could not be detected. Among those cases where the extrapolation results can be compared to data from the literature, worth mentioning are the Hawaiian Islands (with marginal climatic conditions in leeward coastal areas of Honolulu, Lana'i, Kaua'i and Hawai'i), Wake Island, the Republic of Kiribati, Fiji, Samoa, and French Polynesia. Indeed, Hawaiian Islands are home of the endemic wild tetraploid *G. tomentosum*, while an unusual wild form of *G. hirsutum* is locally common in Wake Island [21], [69]. The information available on the presence of wild cotton in Kiribati is less clear, with mentions of *G. tomentosum* [70]–[72], and/or another *Gossypium* species (probably *G. hirsutum*) [73]. Among the Pacific islands cited for wild populations of *G. hirsutum*, only Fiji and Samoa do not appear climatically fit for this species according to our extrapolation; however, this can be related to the rarity of *G. hirsutum* var. *taitense* Roberty in both archipelagos [74].

The excellent correspondence between areas potentially favorable to wild forms of *G. hirsutum* and the actual distributions of wild tetraploid species (*G. hirsutum* itself, *G. mustelinum*, *G. barbadense*, *G. darwinii* and *G. tomentosum*) provides a very interesting example of ecological niche conservatism in evolution [75]. In the present case, it constitutes a further confirmation that the model derived from our Caribbean and TWC population sample is accurate, and indicates that the main driver of tetraploid cotton radiation was geographic isolation, not environmental specialization.

### Potential distribution of *Gossypium hirsutum* in Mesoamerica and the Caribbean at the Last Glacial Maximum

**Figure 5:**
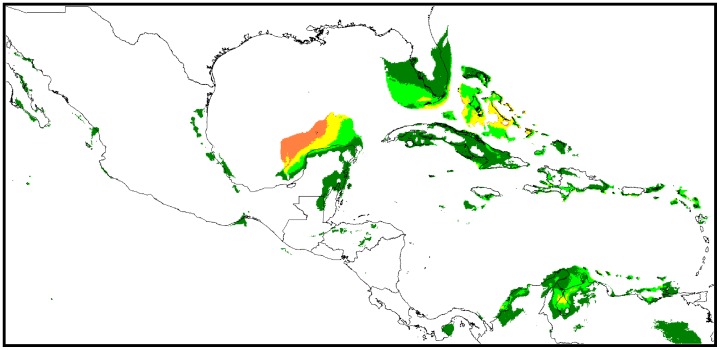
Potential distribution of *G. hirsutum* during the Last Glacial Maximum (21,000 BP). Potential distribution of *G. hirsutum* in the Caribbean and in the Gulf of Mexico, extrapolated according to the MIROC climatic model for LGM. (Note that sea level differences at LGM explain variation with modern sea shore delimitation).

Figure 5 presents the potential distribution of ‘truly wild’ *G. hirsutum* for LGM climates, i.e. about 21,000BP. Sea level was ca. 125 m lower at that time, and rose markedly from 17,000 to 7,000 BP [76]. According to the MIROC model, most areas where ‘truly wild’ cotton populations are found under modern climates were only slightly less favorable at LGM. A few very small favorable areas, such as the one along the shores of Tamaulipas, were at best marginally fit for *G. hirsutum*. In contrast, three areas show a considerable extension at LGM, with many more favorable emerged lands: (i) northern Yucatán, (ii) southern Florida, the Bahamas and Virgin Islands, and (iii) the western shore of Venezuela and a small area on the northeastern Colombian shores. On the whole, *G. hirsutum* distribution was probably much more extended in the Caribbean and in the Gulf of Mexico during late Pleistocene and early Holocene. The main picture is consistent with the hypothesis of Fryxell [24] that Pleistocene shoreline movements were decisive in the evolution and adaptation of tetraploid cottons.

Further south, in equatorial and southern America, LGM climatically favorable areas appear essentially similar to modern ones, except for the Brazilian Northeast, which was less favorable (Figure S2) than in modern times (Figure 4). A few marginally favorable areas may have existed along the Mexican Pacific shores (Figure 5). In the Pacific Ocean, the situation appears similar to the modern one (not shown).

### Genetic characterization of ‘truly wild’ cotton and their feral neighbors

**Figure 6:**
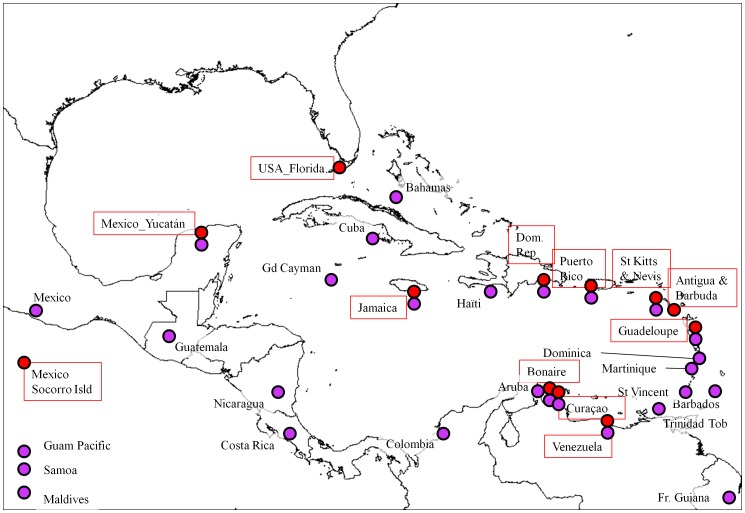
Distribution of the populations of perennial *G. hirsutum* sampled for the SSR-based genetic analysis. Samples include truly wild (TWC) and feral perennial populations in Mesoamerica and the Caribbean. TWC populations are shown as red dots and feral populations are shown as purple dots. Twelve locations where TWC were identified are labeled in red frame. All except USA/Florida, Antigua and Socorro Islands, are also represented by feral specimens, while 18 additional locations had only feral specimens. See Table S1 and Figures S1 for details and precise localizations of the accessions in the 11 sites with TWC populations (Socorro Islands not shown).

The 42 TWC accessions from 11 different (2 for Mexico alone) countries (Figure 6, Table 1 and Table S1, Figure S1) ensure a good representation of the geographical range of truly wild *G. hirsutum* populations as described above; although several similar populations could not be sampled. It appeared very early in the analysis that these TWC populations, including the ‘yucatanense’ population of Yucatán as well as those from diverse places in the Caribbean, showed no racial or geographic differentiation, so we have pooled them in the following presentation. In our sample the ‘feral’ group was represented by 53 ‘Marie-Galante’ accessions of northern South America and the Caribbean and by 15 other accessions (‘punctatum’ from Yucatán, other races from Mesoamerica, and un-ascribed material, Table 1).

SSR statistics are detailed in Table S2. In total, 204 alleles were coded over the 111 accessions and 26 SSR markers, ranging between 3 (HAU2861) and 19 (BNL3103) alleles per SSR. *He* values varied among markers, confirming previous results [26]. Unique alleles amounted to 37 in the feral ‘Marie-Galante’ group (53 accessions) and 43 in the TWC group (42 accessions). *He* shows only limited differences between the different races/categories (Table S3); globally it averages 24.2%, more than usually observed in cultivated cotton (between 5 and 15% under field conditions, but nil in the case of our cultivated control). *He* is slightly higher in wild accessions (28.2%) as compared to feral ones (22.0%). The genetic dissimilarity was also higher within the TWC group (D = 0.51) than within the feral group (D = 0.38) (Table S4).

**Figure 7:**
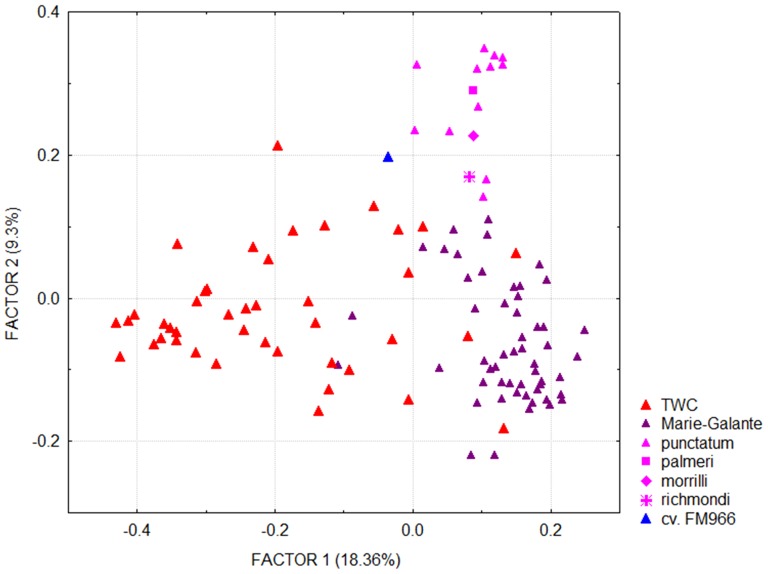
Principal coordinates analysis (PCA) on SSR data in truly wild (TWC) and feral *G. hirsutum*. PCA based on the similarity matrix for 26 SSR markers and 111 accessions represented according to their racial assignation. Factor 1 separates TWC from feral cottons and Factor 2 separates race ‘Marie-Galante’ from other feral cottons.

Both distance-based methods implemented with DARwin, NJ classification (Figure S3) and principal coordinate analysis (Figure 7), separate TWC from feral accessions (first axis in the PCA, Figure 7, and basal branching in NJtree, Figure S3). Within the feral group the analyses further distinguished two subgroups. The first one includes 48 of the 53 accessions of race ‘Marie-Galante’ and the second one includes 17 accessions, 10 of race ‘punctatum’ from Mexico/Yucatán, 6 others (from races ‘morrilli’, ‘palmeri’ and ‘richmondi’ and 3 unassigned), as well as the modern cultivar. Thus, this clustering, which suffers only few exceptions, appears essentially to reflect domestication status (wild vs. feral), and secondarily race. In the nine locations that could be sampled for both feral and TWC accessions (Figure 6), the different analyses clearly indicated that wild/feral status was better than geographical distribution in determining genetic proximity among samples. For example, the TWC from the Atlantic shores of the Lesser Antilles (Guadeloupe, St Kitts and Antigua) were genetically much closer to TWC from northwestern Yucatán, distant by over 2,000 miles, than they were to the feral cottons of the same islands. The genetic relationship among feral cottons is not determined by geographical proximity either: for example, the six ‘Marie-Galante’ accessions from the island of Guadeloupe are not grouped in the same ‘Marie Galante’ branch of the dendrogram (Figure S3B).

**Figure 8:**
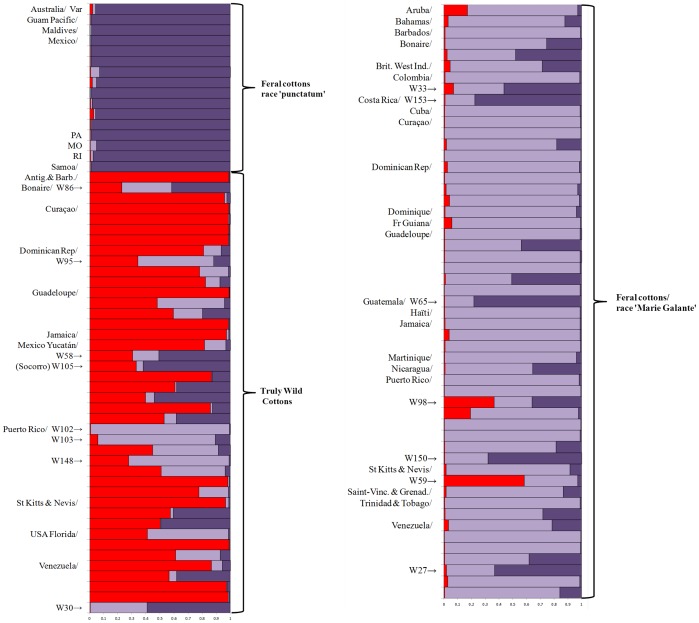
STRUCTURE plot of 111 perennial cottons of *Gossypium hirsutum* with K = 3 clusters. The y-axis shows the proportion membership to the cluster (three clusters depicted in light purple, deep purple and red). Each horizontal bar represents a single accession. The accessions are arranged according to their domestication status and, for feral accessions, their racial assignation, and then alphabetically per country of origin. Fourteen questionable cases (membership to cluster <33%) are indicated with their ‘W’ accession numbers as detailed in Table S1 (see also comments in the main text). Within cluster ‘feral cotton/punctatum’, MO, RI, PA, and Var refer to ‘morrilli’, ‘richmondi’, ‘palmeri’ and modern cultivar, respectively.

The STRUCTURE analysis and ΔK method of Evanno [51] were fully consistent with the two previous ones, clustering the 111 accessions into either two or three clusters, both with high ΔK values (>1000) (Figure S4). Using K = 2 separated TWC from feral cottons (not shown). Using K = 3 further partitioned the feral group in two sub-groups. In Figure 8, we have organized our sample according to the same criteria inferred from both PCA and NJ analyses, but based on field observations: ‘truly wild’ vs. feral, and feral accessions assigned to ‘Marie-Galante’ vs. other feral accessions.

The 42 accessions from TWC populations form a fairly homogenous group (Figures 8 and S3) with an average 67% membership. Only few discrepancies were observed, whereby three accessions had very low (<5%) likelihood of membership to this cluster: W30 (acc. AS0340) from Venezuela, W102 (acc. BPS1240) and W103 (acc. BPS1247) from Puerto-Rico. These accessions were probably wrongly assigned due to an error in collection (although passport data are unambiguous) or a mixture at some stage of multiplication. For a few other TWC assignations, the possibility of *in situ* hybridization cannot be dismissed, as they show an important level of admixture (<50% membership to TWC): W105 (acc. INC035) from Socorro Island, W58 (acc. AS0653) from Yucatán, W86 (acc. BPS1157) from Bonaire, W148 (acc. BPS1239) from Puerto Rico, and W95 (acc. BPS1225) from Dominican Republic. For the latter, the collector mentioned a “different” phenotype with orange pollen and yellow petals [19].

The 53 accessions (22 countries) of race ‘Marie-Galante’ have an average membership of over 81%. This group encompasses the same geographical distribution as the TWC group except for Mexico (Figure 6). Four ‘Marie-Galante’ accessions present higher membership to the other feral group, probably because of wrong race assignation: W153 (acc. CR2000A) from Costa-Rica, W65 (acc. Texas184) from Guatemala, W150 (acc. BPS1243) from Puerto Rico, and W27 (acc. AS0335) from Venezuela. Two ‘Marie-Galante’ accessions, W98 (acc. BPS1230) from Puerto Rico and W59 (acc. AS0681) from St Kitts and Nevis, show high levels of admixture with the TWC cluster and both present unusually high rates of heterozygote SSR, of 64% and 58% respectively; they probably result from an hybridization. Of the two accessions sampled in Colombia, one (W32, acc. AS0435) presents 98% membership to the ‘Marie Galante’ cluster while the other one (W33, acc. AS0437) presents some admixture. It is noteworthy that the latter, W33, belongs to a series of ‘Marie-Galante’ from Northern Colombia, near Barraquilla, described by Ano and Schwendiman [42] as “híbrido nativo; offspring of ancient deliberate crossings between local spontaneous ‘Marie-Galante’ and commercial varieties of *G. hirsutum* or *G. barbadense*.”

Lastly, the mostly ‘punctatum’ branch of feral cottons presents the lowest level of admixture (>97% membership). With 16 accessions, this group includes 12 ‘punctatum’ accessions [9 from inland-Yucatán (as opposed to TWC from the northern shores of Yucatán, - 1 from Maldives in the Indian Ocean (W181, acc. KLM1872), - 2 from Pacific islands (W157, acc. TX-0997 from Guam, W108 acc. TX-1295 from Samoa)], one representative of Mexican races, ‘morrilli’, ‘richmondi’ and ‘palmeri’, and the modern cultivar (FM966) from Australia. The homogeneity of this group indicates that the genetic differentiation among Mexican races [26] is negligible as compared to their divergence from both ‘Marie-Galante’ and TWC populations.

### Distinctiveness of wild and feral populations

The genetic structure observed in a broad collection of cottons representing a vast region of Mesoamerica, Central America, the eastern Caribbean, and even Pacific islands (110 accessions, from 29 different countries or islands) demonstrates that the major driver organizing this collection is the status, feral or wild, of the cotton population, rather than any geographical factor. Thus, ENM and genetic analyses converge in discriminating TWC populations from feral populations, as assessed in our categorization exercise. We can conclude definitively that, not only do ‘truly wild’ populations of *G. hirsutum* still exist, but they are ecologically and genetically distinct, occupying a narrow and well defined habitat. Their genetic distinctiveness and homogeneity invalidate any racial or specific distinction among TWC populations of *G. hirsutum*, such as their classification into race ‘yucatanense’ in northern Yucatán and into race ‘punctatum’ in the Caribbean. *A fortiori*, our results do not support any particular status for the wild perennial cottons from the Dominican Republic, which were the most ‘inland’ collections among our TWC samples (see Figure S1). These wild cottons had been given racial status (*G hirsutum* race ‘ekmanianum’) or specific status, as *G. ekmanianum* Wittmack [56], [77], and they had even been proposed as a new species by Wendel [78], and other authors, of genome AD_6_ (other 5 tetraploid species being denoted as AD_1_ to AD_5_). Our results do not support such proposals as these specimens fall within the overall range of TWC accessions (Figure S3B). Instead, they unambiguously validate the opinion of Schwendiman and colleagues [19], [27] who recognized their morphological unity, from Yucatán to Florida, the Antilles and Venezuela, grouping them under race ‘yucatanense’.

The genetic and ecological divergence between race ‘yucatanense’ *sensu* Schwendiman and feral populations is clearly stronger than the splitting of the latter into two clusters corresponding to (i) races of pure *G. hirsutum* from Mesoamerica, and (ii) the Caribbean and Central American representatives of race ‘Marie-Galante’ resulting from an introgression with *G. barbadense*. This comparison indicates that domestication resulted in a major infraspecific division in *G. hirsutum*. In any case, the low level of admixture between neighboring TWC and feral populations shows the effects of surprisingly strong reproductive barriers and/or very strict ecological adaptation, resulting in very limited gene flow, despite their geographical proximity.

### Distribution and domestication status in *Gossypium hirsutum*

The much stronger differentiation of TWC populations is reminiscent of the study of Stephens [9] who used a similar categorization approach to evaluate the effects of domestication on seed and fiber properties of perennial cottons, well before he formally admitted the existence of ‘truly wild’ populations of tetraploid cottons. As in our ecological and genetic analyses, his “wild” category was clearly the most distinct. Thus, there were highly significant differences in seed grade, seed index and lint index between the wild and feral categories, whereas differences among feral and cultivated categories were much less marked. The morphological, genetic, and ecological proximity between cultivated and feral cottons can be easily explained if they are closely related, i.e. if the latter are still part of the domesticated genepool. This is first suggested by the fact that feral plants show the same geographic patterns of morphological differentiation as cultivated materials [6], [9], [10]. Second, the correlation between the occurrence of feral populations and the cultivation of perennial cotton has been reported by most experts, including Ulloa et al. [10] who observed that feral populations are getting rarer as the cultivation of cotton declines in Mexico. This indicates that most feral populations depend on cultivation of ancient landraces for their perpetuation, following a sink-source dynamics model; in ecological terms, their realized niche is wider than their fundamental niche [79]. This double dependence on man, for their man-made habitat and for their reproduction from cultivated plants, contrasts with the long-term permanency of wild coastal populations of *G. hirsutum*. For example, the wild population of Portland Point in Jamaica was mentioned by Britton in 1908 [57], Schwendiman et al. in 1986 [19] and Stoddart and Fosberg in 1991 [58]. Such cases provide excellent illustrations of the fact that, in its original condition, *G. hirsutum* is a pioneer plant colonizing disturbed coastal habitats, but that “this (habitat) instability is in itself highly stable” and very ancient, so “that the pioneers are simultaneously old residents”, as Fryxell [24] put it. The relationship between extreme aridity and the occurrence of wild cotton is obviously related to the fact that very few other plant species can compete under such conditions, suggesting that our TWC-specific climatic model is fairly representative of its realized niche. Indeed, as stated by Hutchinson [80], even the most mesophytic members of *Gossypium* are intolerant to competition, particularly at the seedling stage. Contrary to feral cotton, the realized niche of TWC populations is narrower than their fundamental niche.

Thus, the present study provides an opportunity to analyze the effect of domestication on the distribution of cultivated perennials, a rarely studied aspect of domestication. Miller and Knouft [81] have analyzed the case of the jocote or purple mombin (*Spondias purpurea* L.), a small fruit tree native from the dry forests of southern Mexico and Central America, and cultivated for its fruit and/or as a fence. They found that the climatic envelope of the wild populations is nested in that of the cultivated forms. In other words, domestication and cultivation mostly expanded the range of the species. Miller and Knouft [81] attributed this expansion to genetic adaptation, discarding the effect of tending cultivated trees, and, more surprisingly, neglecting the effect of the domestication syndrome itself. Indeed, the domesticated purple mombin produces mostly sterile fruits, so it is essentially reproduced from cuttings that grow much faster than seedlings [82], under much less intense competition.

As compared to purple mombin and the majority of perennial fruit crops, *G. hirsutum* differs in its relatively high level of autogamy and endogamy [83]. Domestication has considerably increased the diversity of the species [6], [26] and apparently extended its ecoclimatic range (Figure 3C and D), well beyond the most peripheral and arid habitats of TWC populations. Thus the question remains fundamentally the same: have domestication and selection under cultivation widened the fundamental ecoclimatic envelope of perennial *G. hirsutum* through selection and genetic adaptation? As this envelope is common to feral and cultivated populations, and the feral populations depend on the permanent contribution of cultivated cotton, the most likely answer is that the much wider distribution of these two categories is essentially related to the reduction of competition in cultivated and neighboring disturbed habitats, not to a genetic effect. Furthermore, as the domestication syndrome involves seed characteristics (e.g. seed permeability, hardseededness, dormancy) that are essential for the survival of wild populations, most feral cottons are unable to re-colonize, and persist in, the original habitat of the species. Thus, the apparent paradox is that, although the geographical distribution of perennial *G. hirsutum* has been considerably widened by domestication and cultivation, its niche has been reduced by the loss of reproductive capacity in its natural habitat. In fact, there would have been a true paradox if cultivation, while reducing exposure to both extreme aridity and competition, had increased the competitive potential of *G. hirsutum* in secondary habitats.

### Domestication of *Gossypium hirsutum*

Among the important reasons to study the natural distribution of perennial *G. hirsutum* in Mesoamerica and the Caribbean are the identification of potential areas for the early domestication processes and the comparative characterization of domesticated versus wild cottons. Our distribution maps do not contradict the hypothesis of Brubaker and Wendel [8] of an initial domestication of *G. hirsutum* in northern Yucatán, as this region, home of the most extensive wild populations, indeed corresponds to the largest favorable area (Figure 2A). This has been true not only for the last three millennia under modern climates [53] but very likely also for all the Holocene and even earlier, during the late Pleistocene (Figure 5). On the other hand, our maps also support the views of Sauer [11] on a more diffuse process in space and time, with early lint gathering and even trade preceding regular cultivation. Such a process is consistent with the descriptions of multiform exploitation of different cotton populations by Caribbean natives in early colonial chronicles [9], reminiscent of the domestication processes described by Casas et al. [33]. Clearly, *G. hirsutum* lends itself particularly well to such practices. It is naturally restricted to marginal habitats, where it does not suffer much from competition, but as a pioneer species it could have responded very fast and positively to disturbance by man. Its propensity to cross the boundaries between wild, disturbed and cultivated habitats is still obvious today. We can easily imagine how cotton may have invaded spontaneously the surroundings of fishing communities living close to a natural population. Some basic selection in this new habitat would have steadily brought some improvement, progressively providing the genetic basis for more intense management under managed cultivation, and thereby triggering the domestication process. Once the domestication syndrome was acquired, cultivated forms could not revert to the ‘truly wild’ condition, favoring spatial isolation between the two forms, and in turn further strengthening selection and domestication processes.

However, while the process described above may have taken place both in Mesoamerica and the Caribbean, our genetic data do not favor domestication in the latter area, as Caribbean feral populations appear more closely related to Mesoamerican cultivated and feral cottons than to local TWC populations. Thus, the most likely hypothesis remains that of Brubaker and Wendel [8], with a very early domestication of *G. hirsutum* in northern Yucatán, followed by its progressive diffusion and racial differentiation in all Mesoamerica, then Central America and northern South America. There, race ‘Marie-Galante’ would have developed through introgression with domesticated forms of *G. barbadense*, as hypothesized by Stephens [7], before reaching the Caribbean.

### Conservation of the genetic diversity of *G. hirsutum* and potential interest of wild perennial cottons for breeding

Strategies for the conservation of cotton genetic resources must take into account the relationship between cultivated, feral and wild populations, and the risks of genetic erosion. In the case of the domesticated gene pool, Ulloa et al. [10] have underlined that in southern Mexico *G. hirsutum* perennial cottons survive only as curiosities in garden plots or dooryards, or as occasional feral plants; while attempts at commercial cotton production have been abandoned. In the case of wild cottons, their very ancient habitat is being increasingly threatened, as international tourism covets the same sea-and-sun ecoclimatic niche of dry tropical coasts [19].This point is important in considering the long term *in situ* conservation of perennial cotton *G. hirsutum* populations. Although not considered in this study, the cases of endangered wild *G. barbadense* populations of southern Ecuador/northern Peru, as well as of *G. mustelinum* from northeastern Brazil [84], are similar in ecology and climatic conditions. Only *G. darwinii* from Galapagos is not threatened [85]. The conservation and further plant exploration of wild cottons is important. As highlighted by the results of Liu et al. [28] and Lacape et al. [26], these cottons may have up to 70% unique alleles.

The ecological niche where these wild cotton populations are encountered in Mesoamerica clearly indicates that they represent a great reservoir for genes and alleles related to tolerance to abiotic stresses (water, high temperature or saline stresses). Even though these wild cottons are excellent sources for widening the genetic base for breeding because of their complete interfertility with modern cultivars of *G. hirsutum* [86], this type of material has so far been poorly characterized for its physiological and eco-physiological adaptive traits [87], [88] and rarely exploited in breeding programs [89], [90].

Lastly, a further understanding of the domestication process, through the comparison of the domesticated and wild pools of *G hirsutum*, for example at the transcriptome level [38], as well as for the identification of valuable phenotypic traits [91], [92], can only benefit from an *ad hoc* categorization as attempted in the present study.

## Conclusions

Ocean diffusion and ecological constraints, related to extreme aridity and low levels of competition, best explain the past and current distribution of truly wild populations of *G. hirsutum* restricted to littoral or related habitats, on the shores of the Caribbean Sea and the Gulf of Mexico from Venezuela to Florida, and even as far as Polynesian islands in the Pacific Ocean. The obvious niche conservatism expressed in the strong similarity of the natural habitats of all five allotetraploid species shows that their speciation was essentially driven by the geographic, rather than ecological, isolation of their highly scattered populations.

Our ecological and genetic data consistently support the hypothesis of Brubaker and Wendel [8], indicating that upland cotton domestication was very probably initiated in its largest native population, in northern Yucatán. Cultivated forms then diffused progressively to all the Mesoamerican cultural area, differentiating progressively into the five Mesoamerican races, following a process of geographical and cultural isolation [7]. The diffusion of both New World domesticated cottons, *G. hirsutum* and *G. barbadense*, would have allowed genetic introgression in southern Central America and/or northern South America, resulting in the development of race ‘Marie-Galante’. The close genetic relatedness between ‘Marie-Galante’ and the Mesoamerican domesticated races shows that the introgression process was anterior to the diffusion of domesticated *G. hirsutum* to the Antilles.

Even where domesticated and TWC forms grow in close proximity, they hybridize only sporadically. As a result, the level of genetic divergence between them overwhelms differentiation among domesticated races and/or geographic regions.

Our understanding of plant evolution under domestication is more limited for perennial plants than for seed-propagated annual crops [93]. With their evolution from geographically limited wild populations and their concomitant diffusion and racial differentiation, allowing their establishment under warm temperate climates, the two cultivated tetraploid cottons present interesting parallels with the evolution and adaptation of maize in prehistoric and historic times. The persistence of truly wild populations of both species further increases their interest as unique models for understanding how the genomes of perennials respond to selection pressures operating on the relatively short time scale of the domestication process. The existence of three closely related wild species allows situating this process in the general context of the evolution of allotetraploid cottons from a unique hybridization event, 1–2 million years ago [94].

Wild forms of *G. hirsutum*, with seeds only sparsely covered with short fibers but with adaptation to extreme environmental conditions, contrast with cultivated cotton, with its highly valued long-fibered seeds but adaptation to less demanding ecologies. Owing to the advances of genomics and genome sequencing and the ability to scan the genomes of wild species for new and useful genes, we may now be in a position to unlock the genetic potential of the wild germplasm resources of crop plants [91], [92], including cotton. The sequences of the two diploid species with genomes closest to the constitutive genomes of tetraploid cottons, the genome D of *G. raimondii* and the genome A of *G. arboreum*, have already been published [95], [96]; and the sequencing of the AD genomes of *G. hirsutum* and *G. barbadense* is underway. It should be a relatively easy step now to systematically scan wild germplasm for useful genetic variants. However, this presupposes that *ex situ* collections are adequate, accessible and safe, and that *in situ* preservation efforts are effective in safeguarding material not yet in gene banks, and still evolving in the field. This work should facilitate the development not only of efficient strategies for exploiting cotton diversity for crop improvement, but also of strategies for its long-term conservation.

## Supporting Information

Figure S1 **Maps of the 11 locations/islands where truly wild cottons (red dots) were sampled for the SSR-based genetic analysis (Socorro Islands not shown).** Purple dots represent sampling locations for feral populations in the vicinity. The comments attached to each map are extracted (and translated from French) from collecting reports as indicated.
(DOC)

Figure S2 **Potential distribution of** ***G. hirsutum*** **during the Last Glacial Maximum (21,000 BP) in South America, extrapolated according to the MIROC climatic model.**
(DOC)

Figure S3 **Unrooted neighbor-joining tree based on dissimilarities between 110 perennial, and one cultivated, accessions of** ***Gossypium hirsutum*** **based for 26 SSR markers.**
(DOC)

Figure S4 **Prediction of the best value of K (ΔK method of Evanno et al., 2005), from K = 2 to 5 clusters, from the STRUCTURE analysis of 111 perennial cottons of** ***G. hirsutum*** **.**
(DOC)

Table S1 **Detailed records of the 111 accessions used in the SSR-based genetic analysis.**
(DOC)

Table S2 **List of 26 SSR markers, their chromosome localization on the consensus map (Blenda et al., 2012,** ***PlosONE*** **0045739), and summary statistics (as calculated over 111 accessions): total number of alleles,** ***He*** **value as expected heterozygosity, numbers of alleles and unique alleles within groups (MG = race ‘Marie-Galante’, PU = race ‘punctatum’, TWC = truly wild cottons).**
(DOC)

Table S3 **Mean observed heterozygosity (in %) of 26 SSR markers as observed among 110 perennial accessions of** ***G. hirsutum*** **(cultivated variety FM966, as 111^th^ accession was 100% homozygote).**
(DOC)

Table S4 **Mean dissimilarities within and between groups of 110 perennial accessions of** ***G. hirsutum*** **.** MG, PU and TWC refer to ‘Marie-Galante’, ‘punctatum’ and truly wild cottons.
(DOC)
