# Supplementary material for: Distribution and Differentiation of Wild, Feral, and Cultivated Populations of Perennial Upland Cotton (Gossypium hirsutum L.) in Mesoamerica and the Caribbean
Source: PLoS One. 2014 Sep 8;9(9):e107458. doi: 10.1371/journal.pone.0107458 (PMC4157874; doi:10.1371/journal.pone.0107458)
Supplement: Figure S1 — Maps of the 11 locations/islands where truly wild cottons (red dots) were sampled for the SSR-based genetic analysis (Socorro Islands not shown). Purple dots represent sampling locations for feral populations in the vicinity. The comments attached to each map are extracted (and translated from French) from collecting reports as indicated. (DOC) [file pone.0107458.s001.doc]

**Coppens and Lacape, “Wild, feral, and cultivated upland cotton”**

**Supplementary files (4 Tables and 4 Figures).**

**Figure S1.** Maps of the 11 locations/islands where truly wild cottons (red dots) were sampled for the SSR-based genetic analysis (Socorro Islands not shown). Purple dots represent sampling locations for feral populations in the vicinity.

The comments attached to each map are extracted (and translated from French) from collecting reports as indicated.

| **Bonaire**: FAO/IBPGR prospection of Feb-Mar1985, Schwendiman, Percival, Belot: “We have discovered 2 tiny populations of race yucatanense, one with high risk of extinction due to an urbanization project” | **Curaçao**: FAO/IBPGR prospection of Feb-Mar1985, Schwendiman, Percival, Belot: “.. this unique totally wild population near coastline along narrow small peninsula... soil made of coral debris… habitat may rapidly be threatened” |
| --- | --- |
|  |  |
| **Dominican republic**: FAO/IBPGR prospection of Feb-Mar1985, Schwendiman, Percival, Belot as a complement to prospection of Jan-Feb1980, Ano, Schwendiman : “road Azua-Barahona … remarkable population of primitive cottons in a desertic zone with cactus and other xerophytic vegetation” | **Guadeloupe**: F AO/IBPGR of Jan-Feb1980, Ano, Schwendiman, completed in article Ano et al (1982): “a homogenous population from the Pointe des Châteaux, a dry area with xerophytic vegetation … distinct from race Marie-Galante… its narrow zone of distribution presents some risk of extinction due to tourism” |
|  |  |

| **Jamaican republic**: FAO/IBPGR prospection of Feb-Mar1985, Schwendiman, Percival, Belot: “road to Jackson Bay … only one population of 4 plants belonging to race yucatanense” | **Mexico/Yucatán**: FAO/IBPGR prospection of Feb-Mar1982 of Ano, Schwendiman: “… race yucatanense was seen without any natural interruption from the Rio Lagartos area to the neighborhood of Sisal, on ca 200 kms” |
| --- | --- |
|  |  |
| **Puerto Rico**: FAO/IBPGR prospection of Feb-Mar1985, Schwendiman, Percival, Belot: “race yucatanense (local name algodon brujo) exists in the places Salinas de Guernica as a large population along sandy sealine, and Cabo Bajo” | **Saint Kitts**: FAO/IBPGR prospection of Feb-Mar1982 of Ano, Schwendiman: “a population of very primitive cottons at the periphery of a hotel at Frigate Bay” |
|  |  |
| **Venezuela**: FAO/IBPGR prospection of Jan-Feb1981, Ano, Schwendiman: “ two discontinuous regions of Eastern Venezuela, this first between Rio Chico and Barcelona, including the small island of Piritú, and between Cariaco and Carupano .. xerophytic area … primitive cottons of unknown race” | **Florida**: FAO/IBPGR prospection of Feb-Mar1985, Schwendiman, Percival, Belot: “we were very surprised to find in the Keys, Tavernier Key, Lower Matecumbe, the Everglades (in an unsual mangrove habitat) and Marco Island, … some with high risk of extinction due to urbanization” |
|  |  |

| **Antigua**: FAO/IBPGR prospection of Jan-Feb1980, Ano, Schwendiman : “a single plant escaped systematic uprooting of spontaneous plants (measure to preserve Montserrat Sea Island), located at Half Moon Bay, Marie Galante … type very primitive” (note : assignation to race ‘Marie Galante’ is contradicted by the SSR marker profile) |  |
| --- | --- |
|  |  |
