# Supplementary material for: Distribution and Differentiation of Wild, Feral, and Cultivated Populations of Perennial Upland Cotton (Gossypium hirsutum L.) in Mesoamerica and the Caribbean
Source: PLoS One. 2014 Sep 8;9(9):e107458. doi: 10.1371/journal.pone.0107458 (PMC4157874; doi:10.1371/journal.pone.0107458)

**Coppens and Lacape, “Wild, feral, and cultivated upland cotton”**

**Supplementary files (4 Tables and 4 Figures).**

**Figure S2.** Potential distribution of *G. hirsutum* during the Last Glacial Maximum (21,000 BP) in South America, extrapolated according to the MIROC climatic model


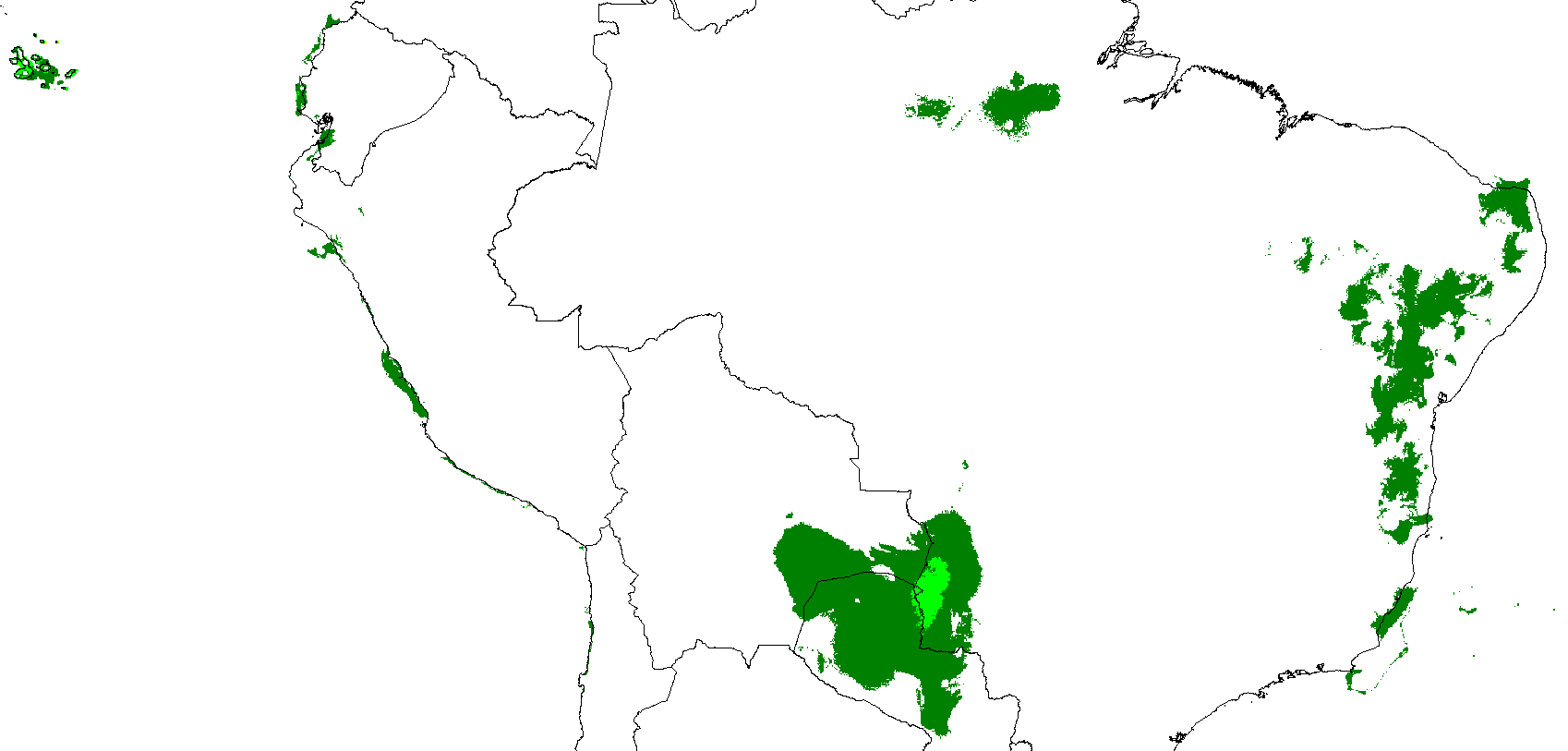

Supplement: Figure S2 — Potential distribution of G. hirsutum during the Last Glacial Maximum (21,000 BP) in South America, extrapolated according to the MIROC climatic model. (DOC) [file pone.0107458.s002.doc]
