# Supplementary material for: Distribution and Differentiation of Wild, Feral, and Cultivated Populations of Perennial Upland Cotton (Gossypium hirsutum L.) in Mesoamerica and the Caribbean
Source: PLoS One. 2014 Sep 8;9(9):e107458. doi: 10.1371/journal.pone.0107458 (PMC4157874; doi:10.1371/journal.pone.0107458)
Supplement: Figure S3 — Unrooted neighbor-joining tree based on dissimilarities between 110 perennial, and one cultivated, accessions of Gossypium hirsutum based for 26 SSR markers. (DOC) [file pone.0107458.s003.doc]

**Coppens and Lacape, “Wild, feral, and cultivated upland cotton”**

**Supplementary files (4 Tables and 4 Figures).**

**Figure S3.** Unrooted neighbor-joining tree based on dissimilarities between 110 wild and feral, and one cultivated accessions of *Gossypium hirsutum* based for 26 SSR markers.

Racial assignations are symbolized in blue for ‘Marie-Galante’, in purple for ‘punctatum’ and related types (MO for ‘morrilli’, PA for ‘palmeri’, and RI for ‘richmondi’), in black for cultivar FM966 (acc. 193), and in red for truly wild cottons.

**A**

**B**

In Figure S3A, unit labels (bold case) correspond to ‘W’ identifier as in Table S1. In Figure S3B, identifiers are FAO country codes (ABW= Aruba, ATG= Antigua-&-Barbuda, AUS= Australia, BES= Bonaire, BHS= Bahamas, BRA= Brazil, BRB= Barbados, BWI=British West Indies, COL= Colombia, CRI= Costa Rica, CUB= Cuba, CUW= Curaçao, DMA= Dominica, DOM= Dominican Rep, ECU= Équateur, , GLP= Guadeloupe, GTM= Guatemala, GUF=French Guiana, HTI= Haïti, JAM= Jamaica, KNA=Saint-Kitts-&-Nevis, MDV= Maldives, MEX= Mexico, MTQ= Martinique, NIC= Nicaragua, PER= Peru, PRI= Puerto Rico, TTO= Trinity-&-Tobago, USA_Pac=Hawaï and Guam, VCT= St Vincent-&-Grenadines, VEN= Venezuela, WSM= Samoa ). Bootstrap percentages (superior to 10) out of 10,000 replications are indicated (normal italics case).
