# Supplementary material for: Distribution and Differentiation of Wild, Feral, and Cultivated Populations of Perennial Upland Cotton (Gossypium hirsutum L.) in Mesoamerica and the Caribbean
Source: PLoS One. 2014 Sep 8;9(9):e107458. doi: 10.1371/journal.pone.0107458 (PMC4157874; doi:10.1371/journal.pone.0107458)

**Coppens and Lacape, “Wild, feral, and cultivated upland cotton”**

**Supplementary files (4 Tables and 4 Figures).**

**Figure S4.** Prediction of the best value of K (ΔK method of Evanno et al, 2005), from K = 2 to 5 clusters, from the STRUCTURE analysis of 111 perennial cottons of *G. hirsutum*.


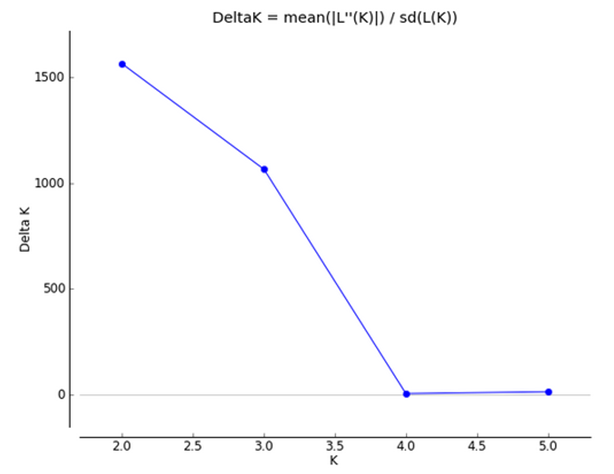

Supplement: Figure S4 — Prediction of the best value of K (ΔK method of Evanno et al., 2005), from K = 2 to 5 clusters, from the STRUCTURE analysis of 111 perennial cottons of G. hirsutum . (DOC) [file pone.0107458.s004.doc]
