# Supplementary material for: Distribution and Differentiation of Wild, Feral, and Cultivated Populations of Perennial Upland Cotton (Gossypium hirsutum L.) in Mesoamerica and the Caribbean
Source: PLoS One. 2014 Sep 8;9(9):e107458. doi: 10.1371/journal.pone.0107458 (PMC4157874; doi:10.1371/journal.pone.0107458)
Supplement: Table S1 — Detailed records of the 111 accessions used in the SSR-based genetic analysis. (DOC) [file pone.0107458.s005.doc]

**Coppens and Lacape, “Wild, feral, and cultivated upland cotton”**

**Supplementary files (4 Tables and 4 Figures).**

**Table S1.** Detailed records of the 111 accessions used in the SSR-based genetic analysis

| **Num in study** | **Race** | **Code CIRAD** | **Acc. Num** | **Synonym** | **Prospection report, year** | **Country** | **Code** | **Site** | **map CIRAD database** |
| --- | --- | --- | --- | --- | --- | --- | --- | --- | --- |
| W8 | MG | 6119 | AS 0134 | TX-1836 | CN 140 (1980) | Antigua & Barbuda | ATG | Half Moon Bay (pointe SE Antigua) | 7 |
| W87 | MG | 7209 | BPS 1161 |  | CN 285 (1985) | Aruba | ABW | Près de la prison d'Oranjestad | 38 |
| W107 | MG | 7629 | TX 1345 |  | USDA | Bahamas | BHS |  |  |
| W17 | MG | 6203 | AS 0220 | TX-1635 | CN 140 (1980) | Barbade | BRB | Mullins Bay | 15 |
| W84 | MG | 7202 | BPS 1154 |  | CN 285 (1985) | Bonaire | BES | Face au Flamingo Beach Hotel | 37 |
| W85 | MG | 7204 | BPS 1156 |  | CN 285 (1985) | Bonaire | BES | 200 m N de Kralendijk | 37 |
| W86 | YU | 7205 | BPS 1157 |  | CN 285 (1985) | Bonaire | BES | Hato au N de Kralendijk | 37 |
| W141 | YU | 7206 | BPS 1158 |  | CN 285 (1985) | Bonaire | BES | Piste menant à Playa Frans | 37 |
| W92 | MG | 7248 | BPS 1200 |  | CN 285 (1985) | BWI, Grand Cayman | CAY | Savannah | 40 |
| W32 | MG | 6397 | AS 0435 | TX-1940 | CN 141 (1981) | Colombia | COL | Galapa (20 km S Barranquilla) | 21 |
| W33 | MG | 6399 | AS 0437 | TX-1708 | CN 141 (1981) | Colombia | COL | 3 km N de Cienaga | 21 |
| W153 | MG | 7525 | CR 2000/A |  |  | Costa Rica | CRI |  |  |
| W156 |  | PI256512 | TX-801 |  | USDA | Cuba | BRA |  |  |
| W80 | MG | 7187 | BPS 1138 |  | CN 285 (1985) | Curaçao | CUW | Rosendaalweg street, W de Willemstad | 36 |
| W81 | MG | 7189 | BPS 1140 |  | CN 285 (1985) | Curaçao | CUW | Bullen Bay | 36 |
| W82 | MG | 7190 | BPS 1141 |  | CN 285 (1985) | Curaçao | CUW | St Willibrordus | 36 |
| W83 | MG | 7193 | BPS 1144 |  | CN 285 (1985) | Curaçao | CUW | Brakkeput | 36 |
| W109 | YU | 7199 | BPS 1151 |  | CN 285 (1985) | Curaçao | CUW | Route de Santa Barbara Beach | 36 |
| W138 | YU | 7196 | BPS 1148 |  | CN 285 (1985) | Curaçao | CUW | 100m E sur Fuik Bay | 36 |
| W139 | YU | 7197 | BPS 1149 |  | CN 285 (1985) | Curaçao | CUW | Route de Santa Barbara Beach | 36 |
| W140 | YU | 7200 | BPS 1152 |  | CN 285 (1985) | Curaçao | CUW | Niewpoort | 36 |
| W2 | MG | 6033 | AS 0039 | TX-1550 | CN 140 (1980) | Dominique | DMA | La Soufrière | 2 |
| W93 | YU | 7255 | BPS 1207 |  | CN 285 (1985) | USA_Florida | USA | Parc des Everglades | 41 |
| W142 | YU | 7254 | BPS 1206 |  | CN 285 (1985) | USA_Florida | USA | Lower Matecumbe Key | 41 |
| W143 | YU | 7257 | BPS 1209 |  | CN 285 (1985) | USA_Florida | USA | Marco Island | 41 |
| W123 | YU | 6602 | AS 0687 | TX-2029 | CN 140 (1980) | Guadeloupe | GLP | Pointe des Chateaux |  |
| W10 | MG | 6172 | AS 0188 | TX-1622 | CN 140 (1980) | Guadeloupe | GLP | Saint-François | 12 |
| W11 | MG | 6174 | AS 0190 | TX-1860 | CN 140 (1980) | Guadeloupe | GLP | Saint-François | 12 |
| W12 | MG | 6179 | AS 0195 | TX-1625 | CN 140 (1980) | Guadeloupe | GLP | Pointe des Chateaux | 12 |
| W13 | MG | 6182 | AS 0198 | TX-1862 | CN 140 (1980) | Guadeloupe | GLP | Pointe des Chateaux | 12 |
| W14 | MG | 6186 | AS 0202 | TX-1629 | CN 140 (1980) | Guadeloupe | GLP | Pointe des Chateaux | 12 |
| W110 | MG | 6167 | AS 0183 | TX-1855 | CN 140 (1980) | Guadeloupe | GLP | Ste-Anne | 12 |
| W62 | YU | 6606 | AS 0692 | TX-2034 |  | Guadeloupe | GLP | Pointe des Chateaux |  |
| W63 | YU | 6609 | AS 0695 | TX-2036 |  | Guadeloupe | GLP | Pointe des Chateaux |  |
| W64 | YU | 6610 | AS 0696 | TX-2037 |  | Guadeloupe | GLP | Pointe des Chateaux |  |
| W157 |  | PI529886 | TX-0997 |  |  | Guam |  |  |  |
| W65 | MG | 6856 | TEXAS 184 |  |  | Guatemala | GTM | Jutiapa, Banque Fort Collins FC 3587 |  |
| W111 | MG | 6215 | AS 0232 | TX-1879 | CN 141 (1981) | French Guyane | GUF | Aouara | 16 |
| W7 | MG | 6093 | AS 0105 | TX-1587 | CN 140 (1980) | Haïti | HTI | 4 km E Jacmel (Meyer) | 6 |
| W88 | MG | 7219 | BPS 1171 |  | CN 285 (1985) | Jamaican Rep | JAM | Entre Portland Cave et Mahoe Gardens | 39 |
| W89 | MG | 7220 | BPS 1172 |  | CN 285 (1985) | Jamaican Rep | JAM | Portland Cottage | 39 |
| W90 | YU | 7221 | BPS 1173 |  | CN 285 (1985) | Jamaican Rep | JAM | Portland Cottage à Jackson Bay | 39 |
| W91 | MG | 7222 | BPS 1174 |  | CN 285 (1985) | Jamaican Rep | JAM | Limite N de Alley | 39 |
| W181 | PU | 7395 | KLM 1872 |  | CN 401 (1988) | Maldives | MDV | Laamu atoll, île Dhabidhoo |  |
| W1 | MG | 6025 | AS 0030 | TX-1547 | CN 140 (1980) | Martinique | MTQ | Sainte-Luce (W canal, bord du stade) | 1 |
| W48 | PU | 6546 | AS 0624 | TX-1978 | CN 197 (1982) | Mexico | MEX | Ignacio Saragoza | 25 |
| W49 | PU | 6547 | AS 0625 | TX-1979 | CN 197 (1982) | Mexico | MEX | 12 km S de Kantunilkin | 25 |
| W50 | PU | 6548 | AS 0626 | TX-1746 | CN 197 (1982) | Mexico | MEX | 15 km W de Kantunilkin | 25 |
| W51 | PU | 6549 | AS 0627 | TX-1980 | CN 197 (1982) | Mexico | MEX | 18 km W de Kantunilkin | 25 |
| W52 | PU | 6552 | AS 0630 | TX-1983 | CN 197 (1982) | Mexico | MEX | 3 km E route de Las Coloradas | 25 |
| W53 | YU | 6553 | AS 0631 | TX-1984 | CN 197 (1982) | Mexico | MEX | Route vers San Felipe | 25 |
| W54 | PU | 6555 | AS 0634 | TX-1986 | CN 197 (1982) | Mexico | MEX | Yordzonot,10 km E Libre Union | 25 |
| W55 | PU | 6557 | AS 0636 | TX-1988 | CN 197 (1982) | Mexico | MEX | Libre Union (Yucatan) | 25 |
| W56 | PU | 6559 | AS 0638 | TX-1990 | CN 197 (1982) | Mexico | MEX | Hacienda Tohoku, route Izamal-Temax | 25 |
| W57 | PU | 6562 | AS 0641 | TX-1748 | CN 197 (1982) | Mexico | MEX | Dzilam Gonzales (Yucatan) | 25 |
| W58 | YU | 6573 | AS 0653 | TX-2002 | CN 197 (1982) | Mexico | MEX | 12 km W de Chuburna Puerto | 25 |
| W116 | YU | 6564 | AS 0643 | TX-1993 | CN 197 (1982) | Mexico | MEX | 2 km E Santa Clara | 25 |
| W117 | YU | 6565 | AS 0644 | TX-1994 | CN 197 (1982) | Mexico | MEX | Santa Clara (Yucatan) | 25 |
| W118 | YU | 6567 | AS 0647 | TX-1996 | CN 197 (1982) | Mexico | MEX | Chabihau | 25 |
| W119 | YU | 6570 | AS 0650 | TX-1999 | CN 197 (1982) | Mexico | MEX | 8 Kms E de Progreso | 25 |
| W120 | YU | 6571 | AS 0651 | TX-2000 | CN 197 (1982) | Mexico | MEX | 8 Kms E de Progreso | 25 |
| W177 | PA | 6523 | AS 0601 | TX-1959 | CN 197 (1982) | Mexico | MEX | PK 118 La Mira-Punta Ixtapa | 24 |
| W178 | MO | 6525 | AS 0603 | TX-1961 | CN 197 (1982) | Mexico | MEX | PK 214,5 Punta Ixtapa-Acapulco | 24 |
| W179 | RI | 6590 | AS 0670 | TX-2018 | CN 197 (1982) | Mexico | MEX | Espinal | 24 |
| W105 | YU | 7368 | INC 035 |  |  | Mexico | MEX | Socorro Island Wild |  |
| W106 | MG | 7600 | TX-1389 |  | USDA | Nicaragua | NIC |  |  |
| W96 | MG | 7274 | BPS 1226 |  | CN 285 (1985) | Puerto Rico | PRI | Playa de Fajardo | 43 |
| W97 | MG | 7276 | BPS 1228 |  | CN 285 (1985) | Puerto Rico | PRI | Ceiba | 43 |
| W98 | MG | 7278 | BPS 1230 |  | CN 285 (1985) | Puerto Rico | PRI | Guayama | 43 |
| W99 | MG | 7279 | BPS 1231 |  | CN 285 (1985) | Puerto Rico | PRI | Ponce, aéroport de la Mercedita | 43 |
| W100 | MG | 7281 | BPS 1233 |  | CN 285 (1985) | Puerto Rico | PRI | SW de Yauco (autoroute) | 43 |
| W101 | MG | 7284 | BPS 1236 |  | CN 285 (1985) | Puerto Rico | PRI | Bahia de la Ballena | 43 |
| W102 | YU | 7288 | BPS 1240 |  | CN 285 (1985) | Puerto Rico | PRI | Salinas de Guanica | 43 |
| W103 | YU | 7295 | BPS 1247 |  | CN 285 (1985) | Puerto Rico | PRI | Bahia Salinas (près Cabo Rojo) | 43 |
| W104 | MG | 7296 | BPS 1248 |  | CN 285 (1985) | Puerto Rico | PRI | SW d'Arecibo (autoroute 22) | 43 |
| W147 | YU | 7286 | BPS 1238 |  | CN 285 (1985) | Puerto Rico | PRI | Salinas de Guanica | 43 |
| W148 | YU | 7287 | BPS 1239 |  | CN 285 (1985) | Puerto Rico | PRI | Salinas de Guanica | 43 |
| W149 | YU | 7290 | BPS 1242 |  | CN 285 (1985) | Puerto Rico | PRI | Salinas de Guanica | 43 |
| W150 | YU | 7291 | BPS 1243 |  | CN 285 (1985) | Puerto Rico | PRI | Parguera | 43 |
| W151 | YU | 7292 | BPS 1244 |  | CN 285 (1985) | Puerto Rico | PRI | Bahia Salinas (près Cabo Rojo) | 43 |
| W152 | YU | 7294 | BPS 1246 |  | CN 285 (1985) | Puerto Rico | PRI | Bahia Salinas (près Cabo Rojo) | 43 |
| W3 | MG | 6063 | AS 0074 | TX-1572 | CN 140 (1980) | Dominican Rep | DOM | 1 km E Azua | 5 |
| W4 | MG | 6067 | AS 0078 | TX-1820 | CN 140 (1980) | Dominican Rep | DOM | 36 km NW Azua à Cortès (route de San Juan) | 5 |
| W5 | MG | 6070 | AS 0081 | TX-1822 | CN 140 (1980) | Dominican Rep | DOM | 50 km NW Azua à Bastidas | 5 |
| W6 | MG | 6072 | AS 0083 | TX-1573 | CN 140 (1980) | Dominican Rep | DOM | 58 km NW Azua, Los Bancos | 5 |
| W94 | YU | 7269 | BPS 1221 |  | CN 285 (1985) | Dominican Rep | DOM | Route Azua à Barahona | 42 |
| W95 | YU | 7273 | BPS 1225 |  | CN 285 (1985) | Dominican Rep | DOM | Route Azua à Barahona | 42 |
| W145 | YU | 7259 | BPS 1211 |  | CN 285 (1985) | Dominican Rep | DOM | Route Azua à Barahona | 42 |
| W146 | YU | 7268 | BPS 1220 |  | CN 285 (1985) | Dominican Rep | DOM | Route Azua à Barahona | 42 |
| W9 | MG | 6120 | AS 0136 | TX-1838 | CN 140 (1980) | Saint-Kitts & Nevis | KNA | Zetlands | 8 |
| W59 | MG | 6596 | AS 0681 | TX-2024 | CN 197 (1982) | Saint-Kitts & Nevis | KNA | Half Way Tree | 26 |
| W60 | YU | 6597 | AS 0682 | TX-1755 | CN 197 (1982) | Saint-Kitts & Nevis | KNA | Royal Hotel St Kitts (golf) | 26 |
| W61 | YU | 6599 | AS 0684 | TX-2026 | CN 197 (1982) | Saint-Kitts & Nevis | KNA | Royal Hotel St Kitts (golf) | 26 |
| W122 | YU | 6598 | AS 0683 | TX-2025 | CN 197 (1982) | Saint-Kitts & Nevis | KNA | Royal Hotel St Kitts (golf) | 26 |
| W15 | MG | 6191 | AS 0207 | TX-1866 | CN 140 (1980) | Saint-Vincent & Grenadines | VCT | Kingstown (jardin botanique) | 13 |
| W108 |  | PI530039 | TX-1295 |  | USDA | Samoa |  |  |  |
| W79 | MG | 7183 | BPS 1134 |  | CN 285 (1985) | Trinité & Tobago | TTO | Scarborough (St Andrew) | 35 |
| W78 | MG | 7160 | BPS 1107 |  | CN 285 (1985) | Trinité & Tobago | TTO | 1 km E de San Francique | 34 |
| W18 | MG | 6297 | AS 0315 | TX-1891 | CN 141 (1981) | Venezuela | VEN | Sortie S Rio Chico (route El Guapo) | 18 |
| W19 | MG | 6301 | AS 0319 | TX-1670 | CN 141 (1981) | Venezuela | VEN | Cupira | 18 |
| W20 | MG | 6302 | AS 0320 | TX-1671 | CN 141 (1981) | Venezuela | VEN | Playa Pintada (W Boca Uchire) | 18 |
| W21 | YU | 6303 | AS 0321 | TX-1892 | CN 141 (1981) | Venezuela | VEN | W Boca Uchire, PK 210 | 18 |
| W22 | YU | 6305 | AS 0323 | TX-1893 | CN 141 (1981) | Venezuela | VEN | 2 km avant Clarines (à l'W) | 18 |
| W25 | YU | 6308 | AS 0326 | TX-1895 | CN 141 (1981) | Venezuela | VEN | Ile de Piritu | 18 |
| W26 | MG | 6313 | AS 0331 | TX-1674 | CN 141 (1981) | Venezuela | VEN | Cuesto Colorado (W de Cumana) | 18 |
| W27 | MG | 6317 | AS 0335 | TX-1676 | CN 141 (1981) | Venezuela | VEN | W de Cariaco (PK 495) | 18 |
| W28 | YU | 6320 | AS 0338 | TX-1902 | CN 141 (1981) | Venezuela | VEN | La Esmeralda (entrée village) | 18 |
| W29 | YU | 6321 | AS 0339 | TX-1903 | CN 141 (1981) | Venezuela | VEN | E La Esmeralda (route Carioca-Carupano) | 18 |
| W30 | YU | 6322 | AS 0340 | TX-1678 | CN 141 (1981) | Venezuela | VEN | E La Esmeralda (route Carioca-Carupano) | 18 |
| W31 | MG | 6327 | AS 0345 | TX-1683 | CN 141 (1981) | Venezuela | VEN | Entrée W Los Ajoyros (E de El Pilar) | 18 |
