# Supplementary material for: Distribution and Differentiation of Wild, Feral, and Cultivated Populations of Perennial Upland Cotton (Gossypium hirsutum L.) in Mesoamerica and the Caribbean
Source: PLoS One. 2014 Sep 8;9(9):e107458. doi: 10.1371/journal.pone.0107458 (PMC4157874; doi:10.1371/journal.pone.0107458)
Supplement: Table S2 — List of 26 SSR markers, their chromosome localization on the consensus map (Blenda et al., 2012, PlosONE 0045739), and summary statistics (as calculated over 111 accessions): total number of alleles, He value as expected heterozygosity, numbers of alleles and unique alleles within groups (MG = race ‘Marie-Galante’, PU = race ‘punctatum’, TWC = truly wild cottons). (DOC) [file pone.0107458.s006.doc]

**Coppens and Lacape, “Wild, feral, and cultivated upland cotton”**

**Supplementary files (4 Tables and 4 Figures).**

**Table S2.** List of 26 SSR markers, their chromosome localization on the consensus map (Blenda et al, 2012), and summary statistics (as calculated over 111 accessions): total number of alleles, *He* value as expected heterozygosity, numbers of alleles and unique alleles within groups (MG= race ‘Marie-Galante’, PU= race ‘punctatum’, TWC = truly wild cottons)

| **Marker** | **chrom** | **Nb alleles total** | ***He*** | **MG** | **Unique**  **PU** | **TWC** |
| --- | --- | --- | --- | --- | --- | --- |
| BNL1145 | 2 | 4 | 0.39 | 1 |  |  |
| BNL1350 | 15 | 15 | 0.72 | 4 |  | 4 |
| BNL1513 | 24 | 5 | 0.69 |  |  |  |
| BNL1551 | 16 | 14 | 0.82 | 2 | 1 | 4 |
| BNL1721 | 18 | 11 | 0.77 | 2 |  | 2 |
| BNL2553 | 20 | 5 | 0.54 |  |  | 1 |
| BNL2572 | 4 | 18 | 0.78 | 6 |  | 4 |
| BNL2921 | 1 | 7 | 0.76 | 1 |  | 2 |
| BNL2986 | 16 | 5 | 0.32 | 1 |  | 1 |
| BNL3065 | 16 | 15 | 0.85 | 3 |  | 6 |
| BNL3103 | 25 | 19 | 0.80 | 5 | 1 | 6 |
| BNL3257 | 8 | 12 | 0.82 |  |  | 1 |
| BNL3261 | 12 | 7 | 0.48 |  |  | 2 |
| CIR036 | 22 | 4 | 0.43 | 1 |  | 1 |
| CIR099 | 18 | 5 | 0.57 | 1 |  |  |
| CIR114 | 1 | 4 | 0.20 |  |  |  |
| CIR246 | 14 | 5 | 0.45 | 1 |  | 1 |
| CLU34 (BNL3411) | 11 | 6 | 0.71 |  |  | 1 |
| CLU80 (CIR203) | 6 | 8 | 0.80 | 1 |  | 2 |
| HAU0894 | 10 | 5 | 0.32 | 1 | 1 |  |
| HAU2861 | 15 | 3 | 0.05 | 1 |  |  |
| HAU3261 | 22 | 6 | 0.57 | 2 |  | 2 |
| MUSS532 | 21 | 6 | 0.27 | 2 |  | 1 |
| NAU1246 | 2 | 5 | 0.40 | 1 |  |  |
| pAR851 | 2 | 4 | 0.56 |  |  |  |
| TMB0478 | 5 | 6 | 0.56 |  |  | 2 |
| **Total** |  | **204** |  | **36** | **3** | **43** |
