# Supplementary material for: Distribution and Differentiation of Wild, Feral, and Cultivated Populations of Perennial Upland Cotton (Gossypium hirsutum L.) in Mesoamerica and the Caribbean
Source: PLoS One. 2014 Sep 8;9(9):e107458. doi: 10.1371/journal.pone.0107458 (PMC4157874; doi:10.1371/journal.pone.0107458)
Supplement: Table S3 — Mean observed heterozygosity (in %) of 26 SSR markers as observed among 110 perennial accessions of G. hirsutum (cultivated variety FM966, as 111th accession was 100% homozygote). (DOC) [file pone.0107458.s007.doc]

**Coppens and Lacape, “Wild, feral, and cultivated upland cotton”**

**Supplementary files (4 Tables and 4 Figures).**

**Table S3.** Mean observed heterozygosity (in %) of 26 SSR markers as observed among 110 perennial accessions of *G. hirsutum*.

| **Race** | Nb | feral | TWC | **Total** |
| --- | --- | --- | --- | --- |
| MG | 53 | 26.1 |  | 26.1 |
| PU | 12 | 7.5 |  | 7.5 |
| MO | 1 | 16.0 |  | 16.0 |
| PA | 1 | 16.0 |  | 16.0 |
| RI | 1 | 15.8 |  | 15.8 |
| TWC | 42 |  | 28.2 | 28.2 |
| **Global** | **110** | **22.0** | **28.2** | **24.2** |
