# Supplementary material for: Distribution and Differentiation of Wild, Feral, and Cultivated Populations of Perennial Upland Cotton (Gossypium hirsutum L.) in Mesoamerica and the Caribbean
Source: PLoS One. 2014 Sep 8;9(9):e107458. doi: 10.1371/journal.pone.0107458 (PMC4157874; doi:10.1371/journal.pone.0107458)
Supplement: Table S4 — Mean dissimilarities within and between groups of 110 perennial accessions of G. hirsutum . MG, PU and TWC refer to ‘Marie-Galante’, ‘punctatum’ and truly wild cottons. (DOC) [file pone.0107458.s008.doc]

**Coppens and Lacape, “Wild, feral, and cultivated upland cotton”**

**Supplementary files (4 Tables and 4 Figures).**

**Table S4.** Mean dissimilarities within and between groups of 110 perennial accessions of *G. hirsutum*. MG, PU and TWC refer to ‘Marie-Galante’, ‘punctatum’ and truly wild cottons.

|  | **MG** | **PU** | **TWC** |
| --- | --- | --- | --- |
| **MG** | 0.38 | 0.52 | 0.56 |
| **PU** |  | 0.39 | 0.61 |
| **TWC** |  |  | 0.51 |
